# Human dermal fibroblast senescence in response to single and recurring oxidative stress

**DOI:** 10.3389/fragi.2025.1504977

**Published:** 2025-03-28

**Authors:** Tailynn Y. McCarty, Cathal J. Kearney

**Affiliations:** Department of Biomedical Engineering, University of Massachusetts Amherst, Amherst, MA, United States

**Keywords:** senescence, aging, wound healing, fibroblast, recurring stress

## Abstract

**Introduction:** Aging results in an accumulation of damaged cells, which reduces the health of tissues and their regenerative capabilities. In the skin, there are both internal and external drivers of oxidative stress that result in aging phenotypes. Oxidative stress has been used to model senescence *in vitro*; however, there has been a lack of research determining whether the severity of oxidative stress correlates with senescent phenotypes.

**Methods:** In this work, we compare cellular and secretory responses to a single (500 μM hydrogen peroxide, 2 hours) or recurring dose of hydrogen peroxide (500 μM hydrogen peroxide, 2 hours + 4 × 300 μM hydrogen peroxide each 48 hours). Senescence induction was studied using markers including cell morphology, senescence-associated-beta-galactosidase, absence of apoptosis, and cell cycle inhibition genes. Next, functional studies of the effects of the signaling of these cells were completed, such as vascular potential, keratinocyte proliferation, and macrophage polarization.

**Results:** Fibroblasts exposed to both single and recurring oxidative stress had increased total cell and nucleic area, increased senescence-associated-beta-galactosidase (SABGAL) expression, and they were able to escape apoptosis – all characteristics of senescent cells. Additionally, cells exposed to recurring oxidative stress expressed increased levels of cell cycle inhibitor genes and decreased expression of collagen-I, -III, and -IV. Cytokine profiling showed that the single stressed cells had a more inflammatory secretory profile. However, in functional assays, the recurring stressed cells had reduced vascular potential, reduced keratinocyte proliferation, and increased IL-1β gene expression in unpolarized and polarized macrophages.

**Discussion:** The described protocol allows for the investigation of the direct effects of single and recurring oxidative stress in fibroblasts and their secretory effects on surrounding healthy cells. These results show that recurringly stressed fibroblasts represent a more intense senescent phenotype, which can be used in *in vitro* aging studies to understand the severity of senescent responses.

## Graphical Abstract

**Figure:**
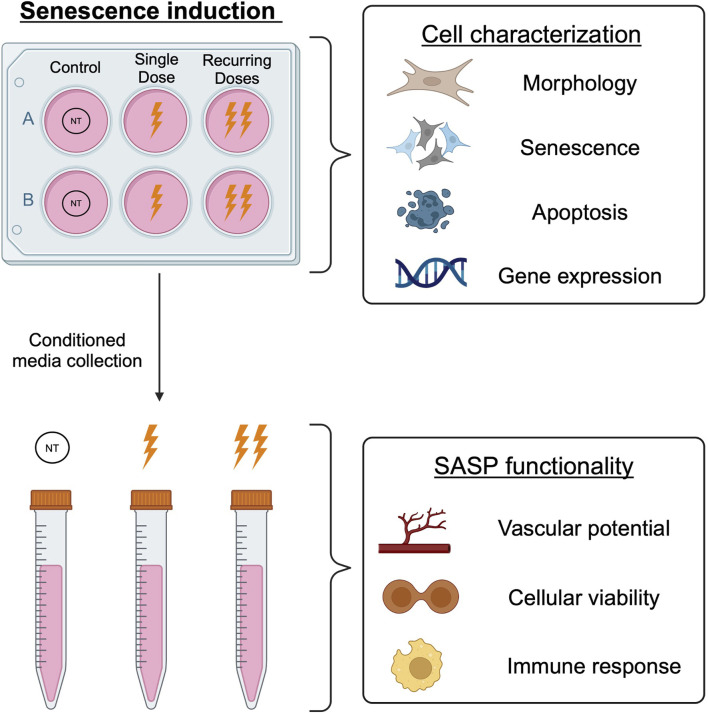
Senescent BJ fibroblasts were generated using two methods of oxidative stress exposure: a single dose and a recurring method with multiple doses. The resulting cells were characterized to investigate the cellular and secretory differences between induction methods.

## 1 Introduction

Aging is an inevitable biological process that results in decline at the cellular and molecular level. Hayflick and Moorehead were the first to discover the limited capacity for cellular division after which cells cease normal proliferation and are in an arrested state known as senescence (Hayflick and Moorhead, 1961). In response to cellular damage, healthy cells will undergo senescence as a protective mechanism and there are some instances where senescence is beneficial for tissue growth and healing. For example, researchers have shown that senescent cells contribute to embryonic growth (Muñoz-Espín et al., 2013) and have been shown to be essential in skin wound healing (Demaria et al., 2014) and regeneration (Feng et al., 2019). In a healthy and optimal environment, these senescent cells are cleared by the immune system and are replaced by healthy cells. By contrast, there is an accumulation of senescent cells within aged tissues. These cells have been correlated with age related diseases such as glaucoma (Liton et al., 2005), type II diabetes (Sone and Kagawa, 2005), and osteoarthritis (Jeon et al., 2017). Currently, researchers are investigating methods of the removal of senescent cells that have accumulated with age; however, this removal has shown both positive and negative effects. For example, Grosse et al. demonstrated that complete removal of p16 positive senescent cells resulted in the reregulation of vessel permeability and subsequent fibrosis, while Wang et al. showed that removal of senescent cells restored healthy functions in aged mice (Grosse et al., 2020; T.-W; Wang et al., 2022).

The skin is a large, dynamic, and complex organ that consists of different layers, each with its own population of cell types with varying functions. Fibroblasts are key cells in the skin that facilitate structural protein production and play a pivotal role in wound healing. Since they play in important role in skin homeostasis and healing, fibroblasts are heavily studied regarding their cellular changes in response to senescence and aging (Wlaschek et al., 2021; Zonari et al., 2023; Rebehn et al., 2023; Low et al., 2021). An important challenge to studying senescent cells *in vitro* is selecting the induction method used to generate them. In addition to replicative methods (i.e., telomere shortening), various types of stressors can be used to induce senescence such as oncogenic activation (Serrano et al., 1997), mitochondrial dysfunction (Wiley et al., 2016), endogenous oxidative stress (Chen and Ames, 1994), and persistent activation of DNA damage (Fagagna et al., 2003), each with their own distinct cellular and secretory phenotypes (Wallis et al., 2022).

As we age, there are increased recurring stressors on the body that cells are accumulatively exposed to. Oxidative stress is particularly interesting for skin studies as oxidative stress is thought to be a pivotal factor in both intrinsic (Stöckl et al., 2006) and extrinsic (Barber et al., 1998) skin aging, including oxidative stress. If senescent cells are not cleared, they are repeatedly exposed to this stress. This suggests that there may be a severity correlation between recurring oxidative stress and senescence severity. By discerning between these senescent cell types and their signaling and roles, we can unlock key understandings in aging and regeneration. Typically, researchers use a single dose of sublethal H_2_O_2_ to generate senescent fibroblasts *in vitro* (Xu et al., 2021; Burnaevskiy, Oshima, and Mendenhall, 2022; Terlecki-Zaniewicz et al., 2019; Facchin et al., 2018; Kiyoshima et al., 2012). However, there is minimal research that investigates the effects of recurring exposure to H_2_O_2_ in dermal fibroblasts. Gerasymchuk et al. demonstrated that as a result of chronic oxidative stress, fibroblasts, had increased in SABGAL expression; however, this study did not directly compare single and recurring exposure. (Gerasymchuk et al., 2022). Given this, it is essential to investigate the cellular differences in senescent cells that are generated from single and recurring oxidative stress. Here, we present a recurring H_2_O_2_ dosing protocol designed to mimic the intermittent *in vivo* chronic exposure to oxidative stress, but over an experimentally feasible time frame. These cells were then compared to senescent fibroblasts that were generated using the standard single dose.

In addition to cellular changes, senescent cells have a very distinct secretome aptly known as the senescence-associated secretory phenotype (SASP), that differs depending on cell type and senescence induction method. This SASP often promotes inflammation due to the upregulated pro-inflammatory factors secreted as a response to senescence induction (Coppé et al., 2010; Basisty et al., 2020; Birch and Gil, 2020). Since cellular differences are observed as a result of different senescence induction methods, we investigated if the SASP of the single *versus* recurring dose oxidative stress induced senescent cells differed. We studied the effect of this SASP on healthy surrounding cells found in skin (e.g., fibroblasts, keratinocytes, vascular cells, macrophages) using conditioned medium experiments in an array of assays.

## 2 Materials and methods

### 2.1 Cell culture

BJ fibroblasts (CRL 2522, ATCC) were cultured in Eagle’s Minimum Essential Medium (ATCC, 30–2003), supplemented with 10% FBS (S1620 Biowest) and 1% Penicillin-Streptomycin (PS) (15–140–122, Gibco). Normal human epidermal keratinocytes (nHEKs) were cultured in Keratinocyte Growth Medium 2 (C-20011, PromoCell) supplemented with the provided supplement pack. Human umbilical vein endothelial cells (HUVECs) were cultured in Endothelial Growth Medium 2 (C-22110, PromoCell) supplemented with the provided supplement pack. THP1 monocytes (TIB-202, ATCC) were cultured in Roswell Park Memorial Institute Medium (RPMI) 1,640 (ATCC, 30–2001) and supplemented with 10% FBS, 1% PS and 0.05 mM 2-Mercaptoethanol. All cells were incubated at 37°C and 5% CO_2_.

### 2.2 Senescence induction

**FIGURE 1:**
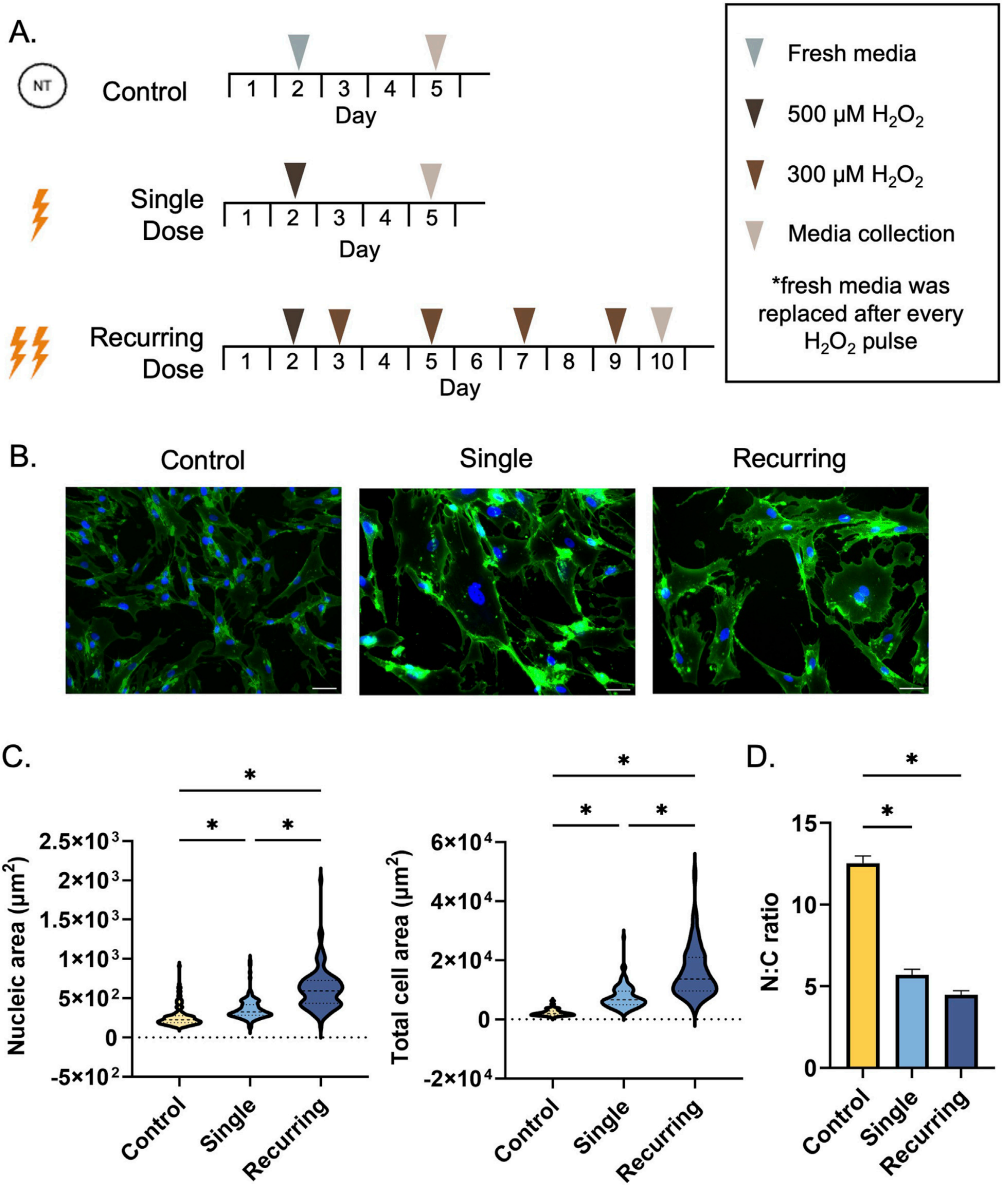
Single and recurring doses of hydrogen peroxide result in morphological changes. **(A)** Cells were exposed to either a single 500 µm dose of H_2_O_2_ (single group) or recurring doses of 300 µm H_2_O_2_ after exposure to the initial 500 µm dose (recurring group). Untreated cells were used as a control. **(B)** A WGA stain was used to observe cellular morphology (plasma membrane = green, nuclei = blue). Scale bar: 100 µm. **(C)** The morphological changes due to senescence induction were quantified by measuring the nucleic and total cellular area using ImageJ (n = 3 per group, with a total of 135 cells analyzed per group). The cytoplasmic area was determined by subtracting the total cellular area from the nucleic area from each cell. Compared to untreated cells, the cellular area significantly increased as a result of single and recurring exposure to hydrogen peroxide (p < 0.001 for both groups). Compared to the control, the nucleus of the cell increased in size (single: p = 0.005, recurring: p < 0.001). Furthermore, there was a severity-dependent response in both cellular and nucleic size with the recurring exposure group resulting in larger cells (p < 0.001) and larger nuclei (p < 0.001) compared to the single exposure group. **(D)** The nucleic to cytoplasmic ratio (N:C ratio) was determined to be reduced in both treated groups (p < 0.001 for both groups). Data was analyzed with a one-way ANOVA test (Tukey’s *post hoc* test). N = 3 for all groups. Significance (p < 0.05) is represented with *. Bars represent mean ± standard error mean (SEM).

Fibroblasts were seeded at a density of 2,000–3,200 cells/cm^2^ depending on the experiment. Passages <12 were used for all experiments. After 48 h, cells in the healthy control group were replenished with fresh media. Both single and recurring groups were washed with PBS and incubated with 500 µM H_2_O_2_ diluted in complete media for 2 h. After incubation, cells were washed with PBS and supplemented with fresh media. The single group was allowed to rest for 72 h before conditioned media collection. For the recurring exposure group, 24 h after the initial 500 µM H_2_O_2_ dose, the cells were incubated with 300 µM H_2_O_2_ for 1 h, washed with PBS, and allowed to rest for 48 h. This process was repeated for a total of four additional pulses after the initial pulse (Figure 1A). 24 h after the last pulse, the conditioned media was collected and used to represent the recurringly stressed group. The culture period for the recurring group was doubled (10 days) to allow sufficient time for repeated doses. Our control and single dose group cultures were stopped at day 5 so that the cells did not become overconfluent.

### 2.3 Investigating senescence induction

#### 2.3.1 Cellular morphology

To quantify the morphological changes after senescence induction, cells were washed with 1X PBS and fixed with 4% paraformaldehyde for 10 min at RT. The plasma membrane was stained with 5 μg/mL wheat germ agglutinin Alexa Fluor 488 (W11261, Invitrogen) according to manufacturer’s instructions. Cells were then incubated with 2 μg/mL Hoechst 33,258 (H1398, Invitrogen) for 5 min at RT protected from light. Five images were taken per flask, with each biological repeat (n = 3; 15 images per biological repeat) consisting of three technical replicates. the Cytoplasmic area was calculated from subtracting the nucleic area from the total cell area.

#### 2.3.2 Senescence-associated-β-galactosidase (SABGAL)

To evaluate cellular senescence, a β-galactosidase assay was performed using a staining kit (9,860, Cell Signaling) according to the manufacturer’s instructions. Cells were then counter stained with 2 μg/mL Hoechst 33,258 for 5 min at RT protected from light. Image collection and experimental design was completed as per the cellular morphology experiment. The total number of cells were quantified per image using ImageJ and then compared to the number of SABGAL positive cells.

#### 2.3.3 Cellular viability and colony formation in response to senescence induction

To investigate cellular viability, fibroblasts were seeded on a 96 well plate and cultured and oxidatively stressed as previously mentioned. On days 3, 4, 5, 6, 8, 10, and 12, 10 µL of CCK8 reagent (Abcam, Ab228554) was added to each group and incubated for 2 h at 37°C. The cellular supernatant was then collected and analyzed according to manufacturer’s instructions. For colony formation, after senescence induction, cells were lifted using trypsin and replated at 200 cells/well in a six well plate and media was changed every 2–3 days. After 12 days (∼6 cell divisions), cells were washed carefully with PBS and fixed in 4% paraformaldehyde for 10 min at RT. Cells were then washed and stained with 0.5% crystal violet in 25% ethanol (40 min at RT), gently washed as to not disturb colonies and allowed to dry at RT. The colonies were the imaged and counted.

#### 2.3.4 Senescence and extracellular matrix associated gene expression

To assess the genetic changes in response to senescence induction and these changes on potential wound healing, the gene expression of senescence markers (*CDKN2A*, *CDKN1A*, and *LMNB1*) and extracellular matrix proteins (*COL1A1*, *COL3A1*, *COL4A1*) were quantified using RT-qPCR. Briefly, RNA was extracted from senescence induced cells using RNeasy Mini kit (Qiagen) and reverse transcribed into cDNA using High-Capacity cDNA Reverse Transcription kit (Invitrogen) according to manufacturer’s instructions. RT-qPCR was performed using iTaq Universal Probes Supermix (Biorad) on a BioRad CFX96 Real-Time PCR System. All Taqman probes were ordered from Thermo Fisher and the catalog numbers can be found in the Supplementary Material. Relative expression was determined using the ΔΔCt method and normalized to housekeeping gene peptidylprolyl isomerase A (PPIA). See Table 1 for probe information.

#### 2.3.5 Caspase-3 and cleaved caspase-3 expression

For homeostatic caspase expression, a caspase-3 quantification kit (Abcam, ab39401) was used to quantify expression. Cells were either senescence induced, non-treated, or positively treated for apoptosis. As a positive control for caspase-3 expression, 10 µM Raptinal was incubated with healthy fibroblasts for 1 h at 37°C. The total protein concentration was quantified using a bicinchoninic acid (BCA) assay. Equal concentration of protein per condition was placed into a 96 well plate, mixed with caspase-3 reagents, and incubated at 37°C for 2 h. The plate was read on a spectrophotometer at 400 nm. To investigate the activation of the caspase-3 cascade, after senescence induction, cells were either left untreated or incubated with 10 µM Raptinal solution diluted in media for 1 h to promote apoptosis. The cells were than antibody labeled as described in the following section.

#### 2.3.6 Senolytic treatment and analysis

After senescence induction, cells were treated with the senolytics Navitoclax (ABT-263) (Cell Signaling, 79,381; 1–10 µM) or cocktail (D + Q) of Dasatinib (Sigma, SML2589) and Quercetin (Sigma, Q4951). Three varying combinations of low (L) and high (H) concentrations of D + Q were used as follows: LD = 100 nM, LQ = 10 μM, HD = 200 nM, HQ = 20 µM. All working solutions of senolytics were diluted in dimethyl sulfoxide (DMSO), which was included as a vehicle control. After senescence induction, cells were washed and treated with senolytics diluted to their final concentration in complete medium for 24 h. After treatment, cells were washed and SABGAL stained, Hoechst counterstained, and analyzed as detailed in Section 2.1.2.

#### 2.3.7 Immunocytochemistry (ICC)

Antibody staining was used to investigate specific cellular characteristics in response to oxidative stress. After treatment, cells were fixed using 4% paraformaldehyde for 10 min, washed 3 times, then permeabilized using 0.1% Triton-X for 5 min. Non-specific binding was blocked with 5% normal goat serum in PBS for 1 h. Cells were incubated with antibodies (cleaved caspase-3 [1:400], *γ*H2AX [1:400], Ki67 [1:800], Cell Signaling) overnight at 4°C followed by incubation with secondary antibody (2 μg/mL), Alexa 488 (Invitrogen) for 1 h at room temperature. Finally, cells were washed and incubated with 2 μg/mL Hoechst 33,258 to stain nuclei and imaged using an EVOS M5000 microscope.

### 2.4 Investigating the effects of senescence secretome

#### 2.4.1 Tubule formation and VEGF secretion

To observe *in vitro* capillary formation, Cultrex Reduced Growth Factor Basement Membrane Extract, Type R1 (R&D Systems) was plated and allowed to solidify at 37°C for 1 h. HUVECs (8 × 10^4^ cells) were resuspended in treatment medium, plated upon the Matrigel, and incubated for 16–18 h. Blank media was used as a negative control with completely supplemented HUVEC media used as a positive control. Tubules were imaged and analyzed using Angiogenesis Analyzer plug-in on ImageJ. To investigate whether angiogenic changes were related to vascular endothelial growth factor (VEGF), senescent cells and controls were lysed, and VEGF was quantified using RT-qPCR. Secretory VEGF expression was quantified in senescent conditioned media using a VEGF ELISA kit (DY293B) according to the manufacturers protocols (R&D Systems).

#### 2.4.2 Cell viability in response to senescent secretome

Fibroblasts (3 × 10^3^ cells) and keratinocytes (3 × 10^3^ cells) were seeded on a 96 well plate and allowed to adhere overnight. Cells were then washed with PBS and serum starved overnight. The cells were then incubated overnight with 100 µL of conditioned media from each group. A CCK8 reagent (Abcam, Ab228554) was used as previously mentioned.

#### 2.4.3 Macrophage polarization

Macrophages were differentiated and polarized as previously described (Sridharan et al., 2019; Santarella et al., 2020; 2022). Briefly, THP-1 monocytes (0.5 × 10^6^ cells) were differentiated to macrophages in media supplemented with 20 ng/mL phorbol 12-myristate 13-acetate (PMA) overnight. Fresh growth media was added after removal of differentiation media and cells were allowed to rest for 6 h. This was followed by the addition of fresh growth medium for unpolarized macrophages (M0), 5 ng/mL interferon-γ (IFN-γ) and 100 ng/mL lipopolysaccharide (LPS) for M1 macrophages, and 20 ng/mL of interleukin-4 (IL-4) and interleukin-13 (IL-13) for M2 macrophages. All cells were incubated in polarization medium for 48 h. The cells were then washed, and treated for 24 h with conditioned medium from control cells, or senescent cells exposed to single or recurring oxidative stress. For secretome protein quantification, the conditioned medium from each macrophage group was collected and IL-1β (DLB50) and CCL13 (DY327) concentrations were quantified using an ELISA (R&D Systems) according to manufacturer’s instructions. For gene expression, RNA was collected, transcribed, and processed as previously mentioned in Section 2.3.3. CD68 was used as a pan-macrophage marker while CD80 and IL-1β and CD206 and CCL13 were used as M1 and M2 macrophage markers, respectively. *GAPDH* was used a housekeeping gene for all macrophage gene expression experiments (See Supplementary Material for Taqman probe details). For surface marker expression, cells were washed with PBS and incubated with trypsin for 5 min at 37°C. FACS buffer (5% FBS in PBS) was used to neutralize the trypsin. Cells were then incubated for 20 min on ice with 2.5 µg Fc block (564,219, BD Biosciences) to reduce unspecific binding. Cells were then stained with an antibody cocktail of CD68 (12–0689–42, Invitrogen), CD80 (17–0809–42, Invitrogen), and CD206 (53–2069–42, Invitrogen) for 1 h. Flow cytometry was performed within 1 h on an BD DUAL LSRFortessa. Data was analyzed using FlowJo software (FlowJo, LLC).

#### 2.4.4 Cytokine profiler

The conditioned medium was collected from all groups and 36 cytokines were probed using Proteome Profiler Human Cytokine Array Kit, R&D Systems according to the manufacturer’s instructions.

#### 2.4.5 Statistical analysis

All statistical analysis was carried out on Graphpad Prism 10. Graphs express means ± standard error means (SEM). One-way ANOVA and Tukey’s *post hoc* test was used to compare the significant differences between three or more groups. Results were considered significant if p < 0.05.

## 3 Results

### 3.1 Exposure to oxidative stress causes morphological enlargement

Upon exposure to oxidative stress (Figure 1A), there were significant differences in morphology of the fibroblasts quantified by total cellular area, cytoplasmic area, nucleic area, and the ratio of nucleic to cytoplasmic area (N:C). In response to oxidative stress, the cellular area and nucleic area significantly increased in both the single and recurring exposure groups compared to control cells (Figure 1B). When oxidatively stressed groups were compared to each other, both the total cellular area and the nucleic area was significantly larger in the recurring exposure group (Figure 1C). The N:C ratio was substantially lower in both senescent groups when compared to the untreated control (Figure 1D). Additionally, we observed an accumulation of micronuclei in both groups, but there were none observed in the healthy control groups (Supplementary Material).

### 3.2 The severity of oxidative stress determines senescent phenotype in fibroblasts

**FIGURE 2:**
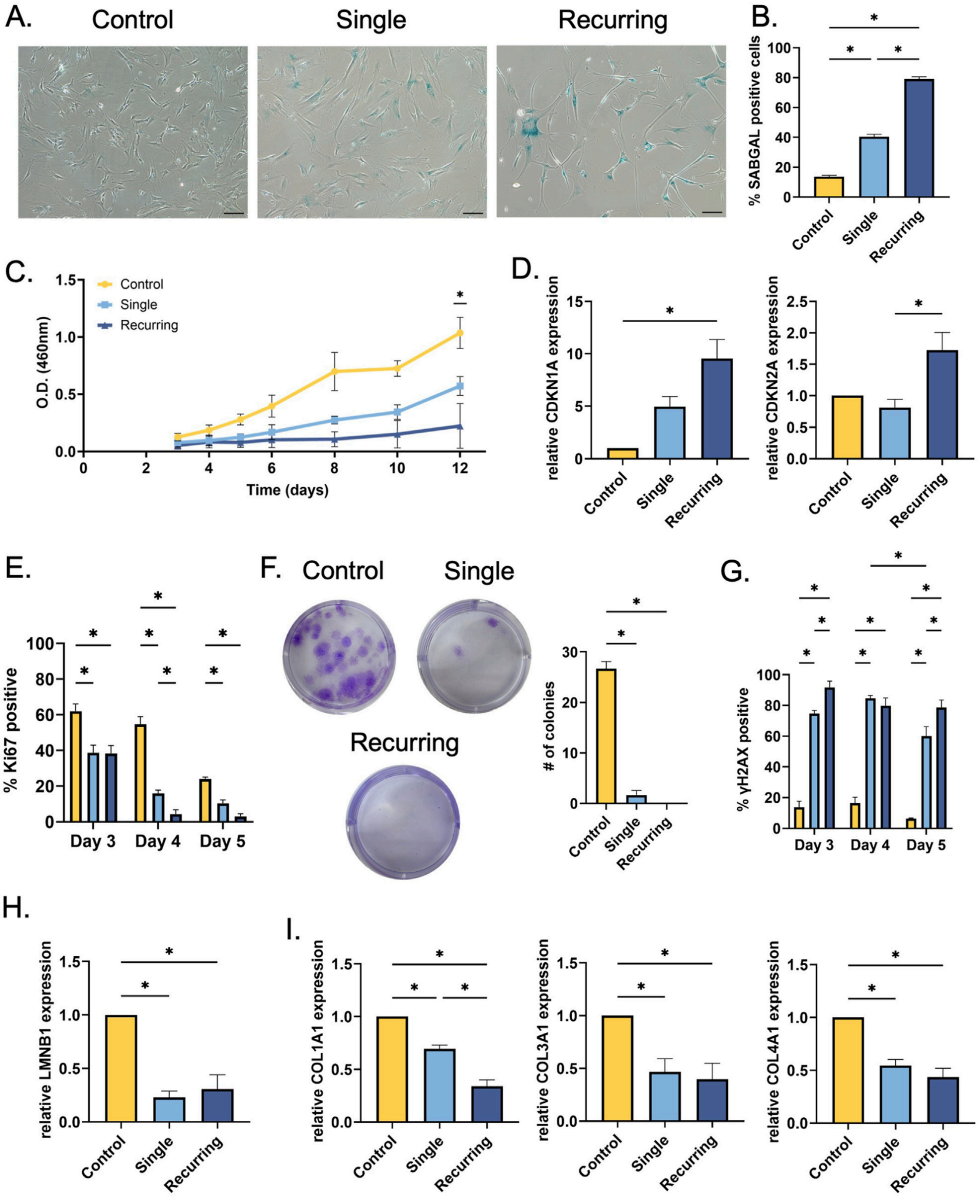
Single and recurring oxidative stress generates different senescent profiles in fibroblasts. **(A)** After senescence induction, SABGAL staining was used to determine whether oxidative stress causes senescence in fibroblasts. **(B)** Cells were counterstained with Hoechst and counted using ImageJ. There was a severity-dependent increase in SABGAL expression, with recurring H_2_O_2_ exposure generating the most SABGAL positive cells (79.0% ± 1.53%) **(C)** After senescence induction, cellular viability was assessed. Over time, cellular viability remained constant in the recurringly stressed group, however, control fibroblasts and single stressed fibroblasts remain viable and continue to grow over time. **(D)** To investigate cell cycle arrest in senescent cells, RT-qPCR was used to determine *CDKN1A* and *CDKN2A* gene expression after senescence induction. The cells exposed to recurring oxidative stress had higher CDKN1A expression (p = 0.005) compared to control, and higher CDKN2A expression compared to cells treated once (p = 0.026). **(E)** Additionally, in response to oxidative stress, there senescent cells lose their proliferative capacity with a significant decrease in Ki67 positive nuclei in both the single and recurring stress groups. **(F)** Further, there was a decrease in colony formation after a single oxidative stress while there were no colonies observed in the recurringly stressed cells (p < 0.001 vs. control). **(G)** After oxidative stress, there was an increasing number of *γ*H2AX positive nuclei. In the stressed groups, the H2AX highest expression occurred on day 3 for the recurring group which is, after the first low oxidative pulse (91.67% ± 4.177%) and on day 4 which is 48 h after the initial induction in the single exposure group (84.67% ± 1.856%**). (H)** A commonly used senescence marker, lamin B1 (*LMNB1*) gene expression was quantified; and both groups exposed to H_2_O_2_ had reduced expression. **(I)** Similarly, collagen-I (*COL1A1*), -III (*COL3A1*), and IV (*COL4A1*) gene expression was also measured after senescence induction. In all treated groups, collagen genes were reduced, with *COL1A1* being significantly reduced in the recurring *versus* singly dosed. Data was analyzed with one-way or two-way ANOVA test (Tukey’s *post hoc* test). n = 3 for all groups. Significance (p < 0.05) is represented with *. Bars represent mean ± standard error mean (SEM).

In response to the oxidative stress, SABGAL expression was increased in both stressed groups compared to healthy cells (p < 0.001). However, the percent of cells that were expressed was significantly increased in the recurring exposure group compared to cells that were exposed to a single dose of H_2_O_2_ (Figures 2A, B). Senescent cells have been shown to cease normal cell cycles and remain in a stagnant, non-proliferating state. We used CCK8 to measure the relative number of viable cells in response to single and recurring exposure to oxidative stress. In the healthy control group and the single exposure group, there was an increase in cellular viability over time, however, with the recurring group, there was no significant difference in cellular viability over time (Figure 2C). This increase in cellular viability can be correlated with cellular growth and overall cellular health. We tested the significance between groups at the final time point and found that there were differences between all three groups (control: p = 0.01, single: p < 0.001, recurring: p = 0.017). To further probe cellular growth cycles, *CDKN2A* and *CDKN1A* gene expression were investigated. There was a significant increase in both geneses for cells that were exposed to recurring oxidative stress (Figure 2D). Ki67 is a marker that is used to investigate the proliferative properties of cells. We have observed a decrease in Ki67 expression in both oxidatively stressed groups over time, showing that senescent cells lose their proliferative capacity as a response to oxidative stress (Figure 2E). On day 4, there was a significant decrease in Ki67 expressing cells *versus* the single dose group, and the percent of proliferating cells in the recurring group was between 0% and 5% up to day 10. Lastly, to verify the loss of growth and proliferative characteristics in senescent cells, we completed a colony formation assay. While control healthy cells formed colonies–confirming their proliferative capacity–there was minimal colony formation in both single stressed senescent cells and no colonies observed in recurringly stressed senescent cells (p < 0.001 vs. control) (Figure 2F).

Previous research has shown that oxidative stress can cause double stranded DNA breaks and previous research has shown that *γ*H2AX is a robust marker to use when investigating DNA damage. We observed a significant increase in *γ*H2AX expression in both senescent groups compared to the control cells that were not exposed to oxidative stress (Figure 2G). Over time, the recurring oxidative stress results in persistent DNA damage up to day 10 (Supplementary Material). However, there was a decrease in *γ*H2AX expression in the single exposure group, suggesting some recovery in this cell population. As an additional cellular senescence marker, lamin B1 (*LMNB1*) was also investigated. In both single and recurring exposed cells, there was a decreased expression in lamin B1 (p = 0.002 and p = 0.003, respectively; Figure 2H).

Since collagen is one of the most abundant proteins in the skin, the effect of oxidative stress induced-senescence on collagen genes was measured. There was a significant decrease in *COL1A1* expression in both groups that were exposed to oxidative stress when compared to controls (p < 0.005 and p < 0.001, respectively), but notably, the recurring group had a significantly lower expression than the single exposure group (Figure 2I). In response to oxidative stress, there was a decrease in both *COL3A1* and *COL4A1* gene expression in both senescent groups compared to control fibroblasts (*COL3A1*: p = 0.037 and p = 0.022, *COL4A1*: p = 0.004 and p = 0.001, respectfully).

### 3.3 Senescent cells escape apoptosis, regardless of the severity of oxidative stress

**FIGURE 3:**
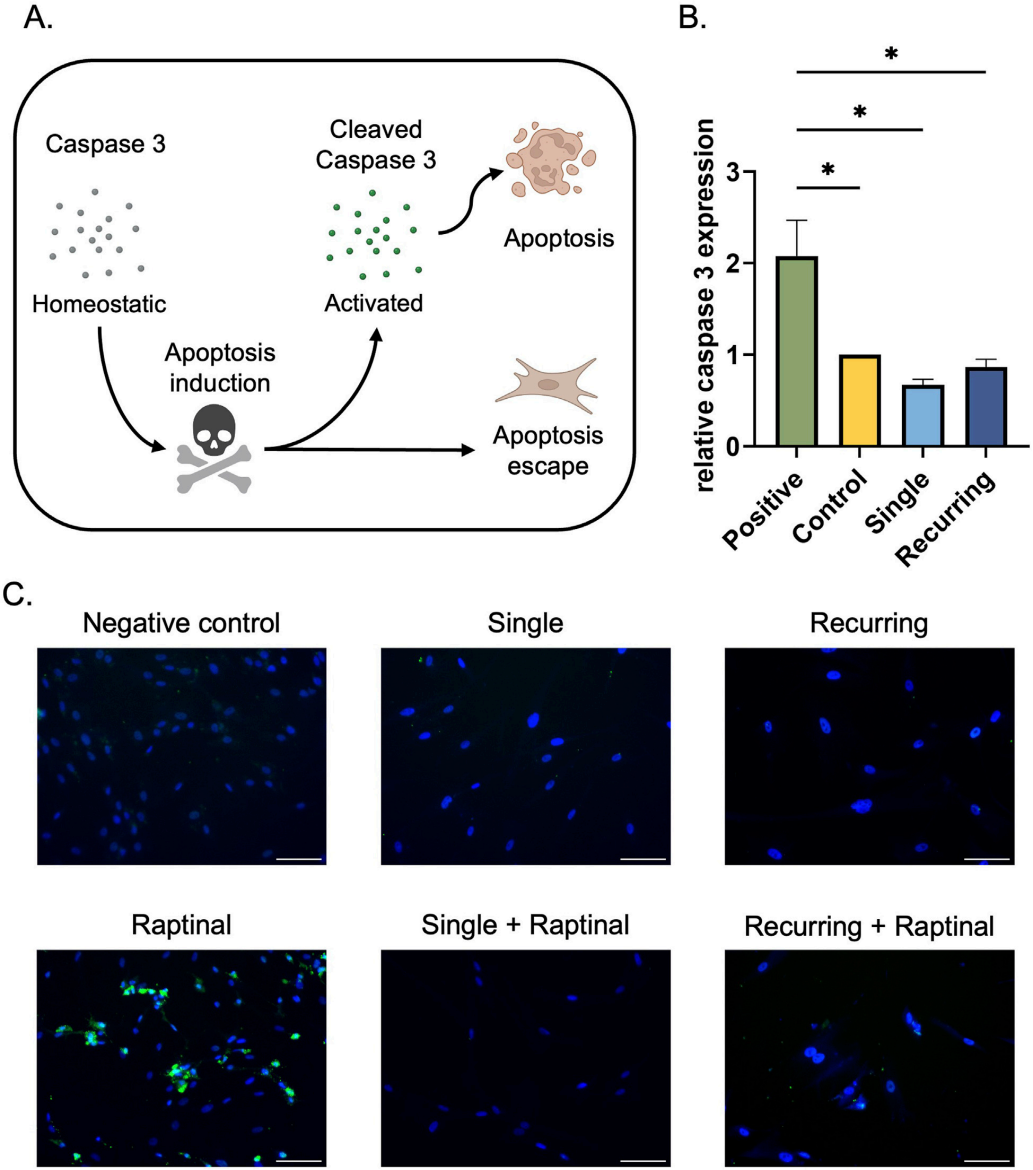
Senescent cells escape apoptosis regardless of caspase-3 expression. **(A)** In the presence of an apoptotic stress, caspase three is cleaved, which initiates the apoptotic cascade and leads to cellular death. Senescent cells have previously been characterized to escape apoptosis via various pathways. **(B)** Fibroblasts exposed to oxidative stress were collected and the homeostatic levels of caspase-3 were quantified. In tandem, 10 µM raptinal was used to induce apoptosis in cells as a positive control. There was a significant increase in caspase three expression in the raptinal group compared to the control and both oxidatively stressed groups. Data was analyzed with one-way ANOVA and Tukey’s *post hoc* test (n = 3); significance (p < 0.05) is represented with *. Bars represent mean ± SEM. **(C)** To determine if the senescent cells were able to evade apoptosis, senescent cells were incubated with 10 µm raptinal for 1 h on day 5 in the single dose group and on day 10 in the recurring dose group. Finally, immunofluorescence staining was performed to assess cleaved-caspase-3 expression. Scale bar: 100 µm.

As a final characterization method, the apoptotic exclusion capabilities of the cells were investigated. Initially, the homeostatic expression of caspase-3 was quantified to determine if there were changes in the apoptotic protein in response to oxidative stress. As a positive control for increased caspase-3 expression, healthy cells were incubated with raptinal, which is a small molecule that is used to induce apoptosis (Vernon et al., 2022; Palchaudhuri et al., 2015). Each group of cells had decreased caspase-3 expression compared to the raptinal-treated group. However, there were no differences between the oxidative stress groups (Figures 3A–C). A well-known characteristic of senescent cells is their ability to escape apoptosis, resulting in eventual accumulation of stressed cells (Hu et al., 2022; Deryabin, Shatrova, and Borodkina, 2021). To determine if the apoptosis cascade was active, we observed cleaved caspase-3 expression, which has previously been used to determine whether the apoptosis cascade has been activated, leading cellular death. Since raptinal induces apoptosis in healthy cells, each senescent group was then incubated with raptinal, and cleaved caspase-3 was investigated. In this instance, we expect non-senescent cells to undergo apoptosis and have increased expression of cleaved caspase-3, whereas cells that have escaped apoptosis will not express cleaved caspase-3. We observed no cleaved caspase-3 expression in senescent cells generated from single or recurring oxidative stress when exposed to raptinal. This suggests that these cells can evade apoptosis (Figure 3C).

### 3.4 Navitoclax treatment reduces SABGAL positive cells *in vitro*

**FIGURE 4:**
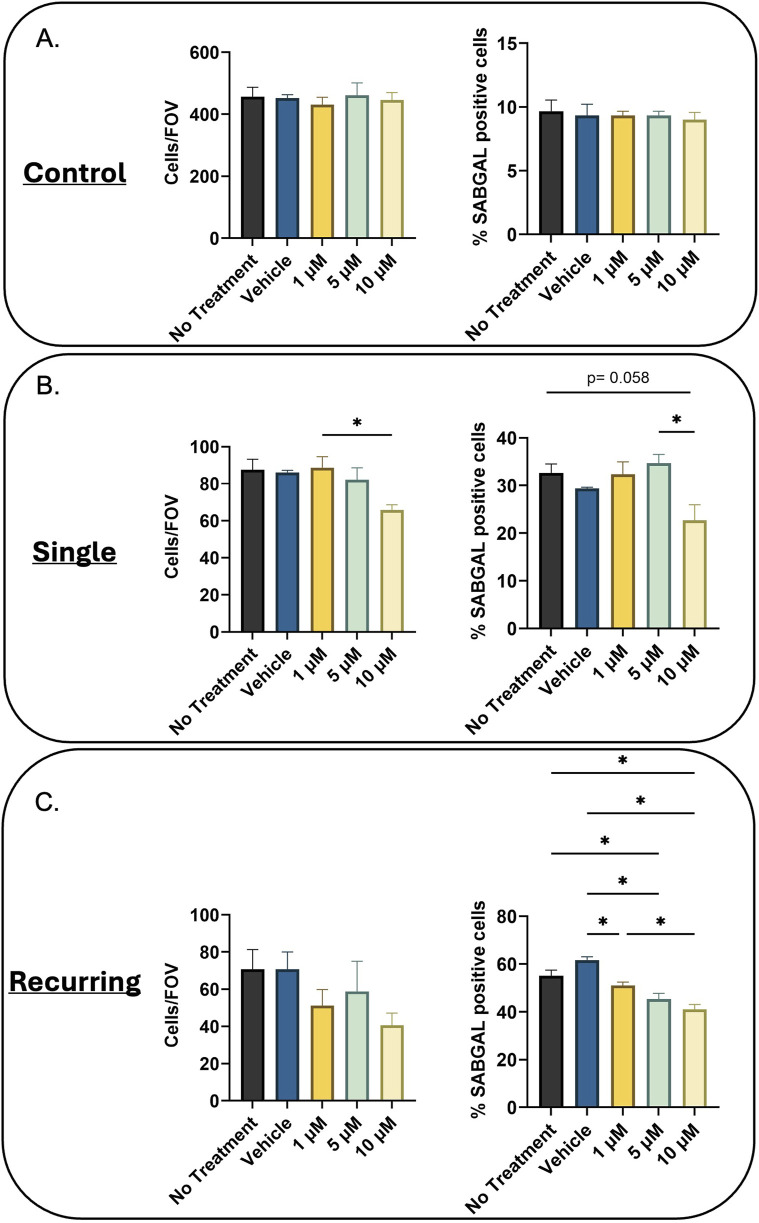
Navitoclax reduces SABGAL positive senescent cells. **(A)** Healthy **(B)** Single oxidatively stressed, and **(C)** recurringly oxidatively stressed fibroblasts were treated with Navitoclax at three concentrations (1, 5, 10 µM) for 24 h. While Navitoclax has no effect on control cells, for the oxidatively-stressed groups, cell number decreased with increasing concentrations of Navitoclax (Cells/Field of View; Cells/FOV). Additionally, there was a downward trend in senescent cells remaining post-Navitoclax treatment (%SABGAL positive cells), which was significant in all treatment groups for the recurring stressed group. Data was analyzed using one-way ANOVA test (Tukey’s *post hoc* test). n = 3 for all groups. Significance (p < 0.05) is represented with *. Bars represent mean ± SEM.

Lastly, we wanted to investigate the effects of senolytics on our generated cells. In the oxidatively-stressed groups, there was a decrease in cell number (not-significant) and after single oxidative stress, 10 µM Navitoclax treatment resulted in a slight downward trend in SABGAL positive cells (p = 0.058). However, in the recurringly stressed group, there was a significant decrease in SABGAL cells regardless of Navitoclax concentration (Figure 4C). Additionally, we tested the cocktail D + Q, which did not demonstrate senolytic efficacy in either the single or recurring stressed cells. (Supplementary Material).

### 3.5 Recurring oxidative stress in fibroblasts results in poor tubule formation

**FIGURE 5:**
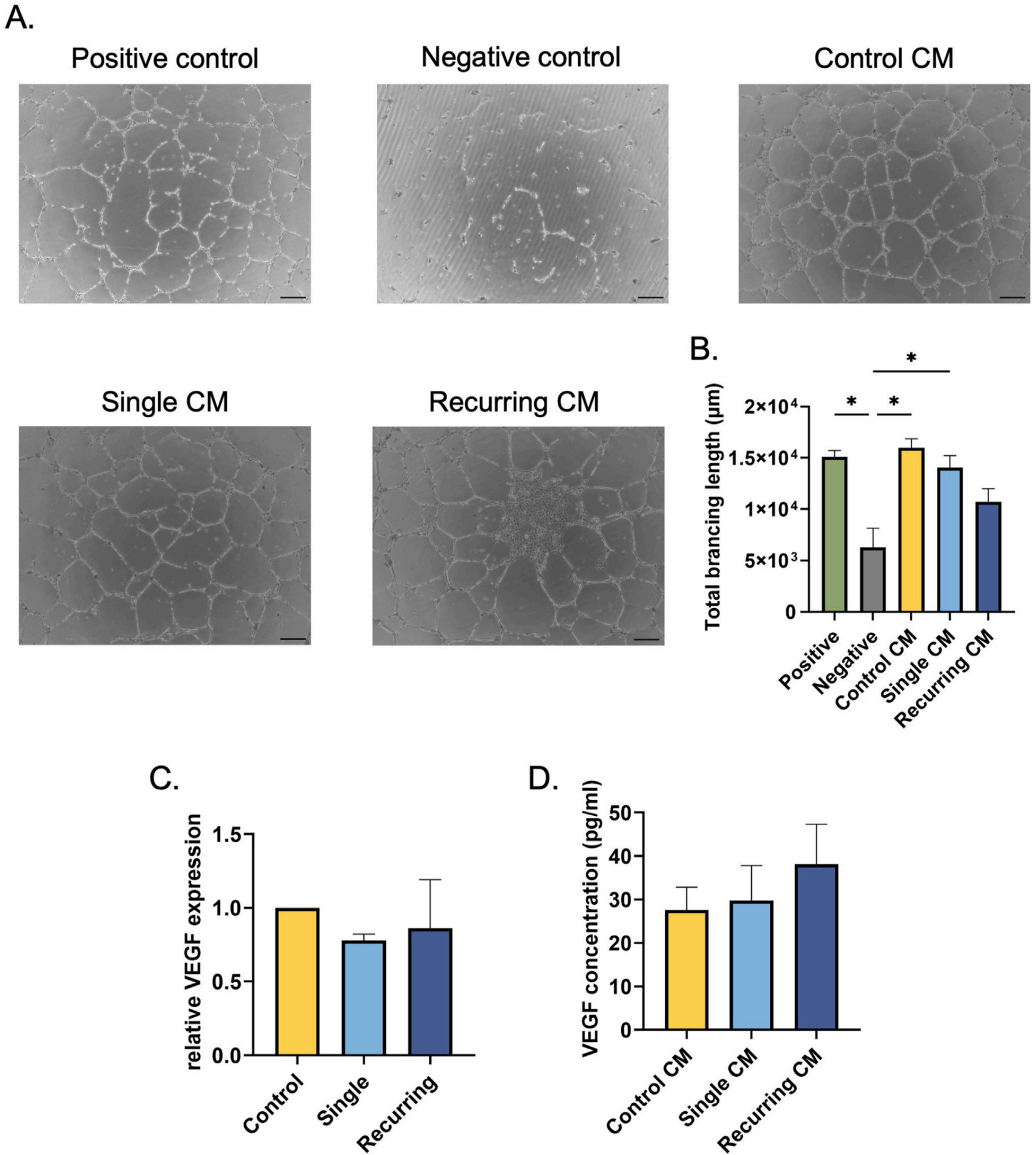
Senescent cells induced via recurring oxidative stress reduced tubule formation. **(A)** HUVECs resuspended in conditioned medium from oxidatively stressed cells were seeded on Matrigel to induce tubule formation. The tubules were imaged after 16–18 h. Scale bar: 100 µm. **(B)** The Angiogenesis Analyzer plug-in in ImageJ was used to quantify total branching length in each group. There was no significant difference between the negative control group and the HUVECS treated with the conditioned medium from recurring dose senescent cells. **(C)** To investigate changes in VEGF expression after oxidative stress, VEGF gene expression was quantified; no differences between healthy controls and oxidatively induced senescent cells from either group. **(D)** Additionally, secreted VEGF was quantified in fibroblasts that were oxidatively stressed and there were no changes in VEGF secretion. Data was analyzed with one-way ANOVA test (Tukey’s *post hoc* test). n = 3 for all groups. Significance (p < 0.05) is represented with *. Bars represent mean ± SEM.

Once the senescent cells were characterized, we wanted to investigate the role that the senescent secretome had on healthy cells using established *in vitro* wound healing assays. First, the effects on vascularization potential were investigated. HUVECS treated with the conditioned medium (CM) collected from untreated control fibroblasts and single-dose induced senescent cells increased the total tubule length compared to the negative control (p = 0.02 and p = 0.009, respectively; Figures 5A, B). However, there was no difference between the negative control and the recurring oxidative stress group. To investigate the role of VEGF on the angiogenic potential, VEGF gene expression and secreted VEGF concentrations were quantified in untreated and stressed groups (Figures 5C, D). There were no differences in VEGF gene expression or secreted VEGF between each group, suggesting that the lack of tubule formation in the recurring senescence group was not mediated by VEGF.

### 3.6 Keratinocyte viability was reduced in response to CM from senescent fibroblasts generated using recurring oxidative stress, while fibroblast viability remained unaffected

**FIGURE 6:**
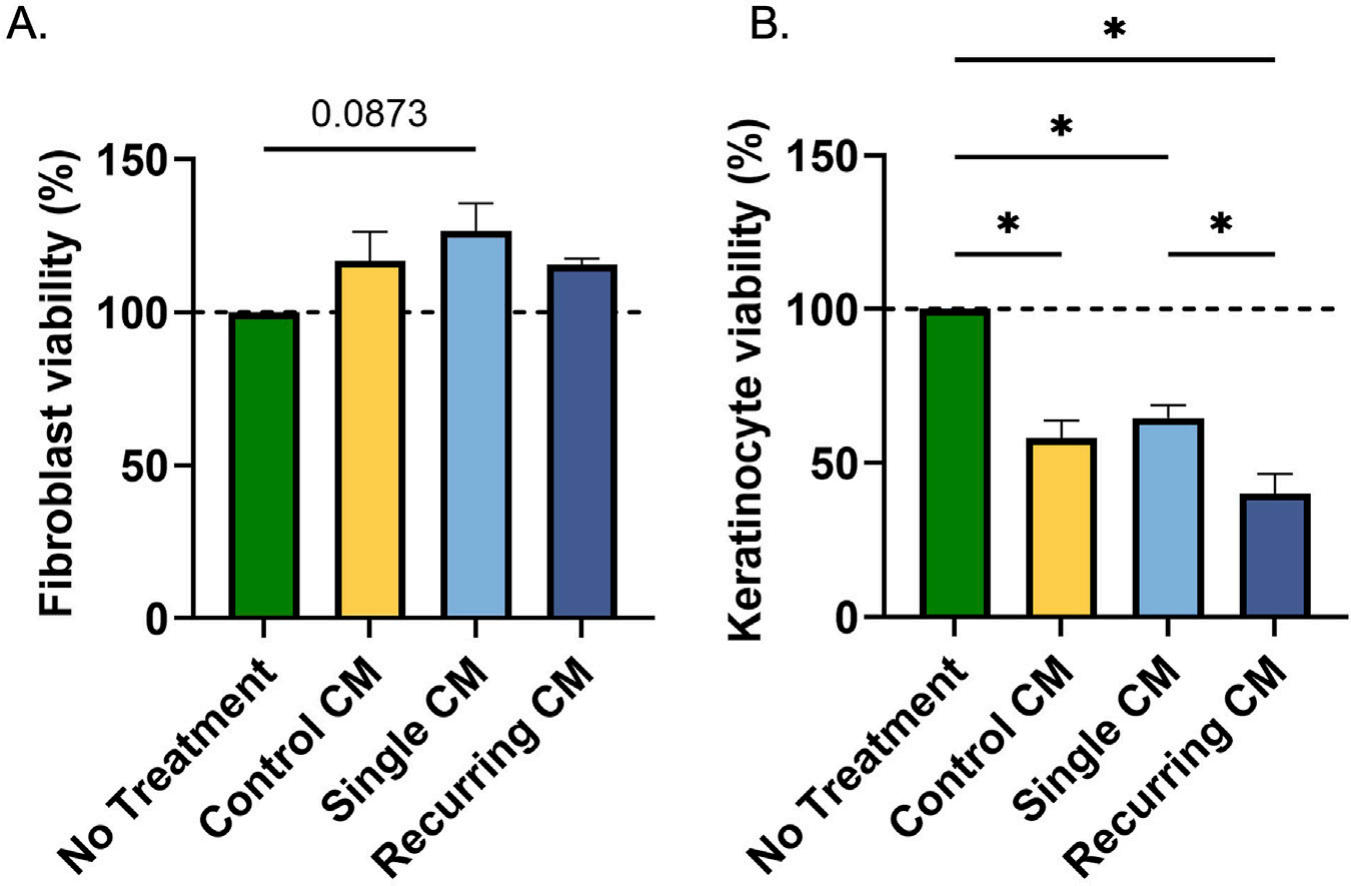
Conditioned medium from oxidatively stressed fibroblast did not affect fibroblast viability but decreased keratinocyte viability. **(A)** Healthy fibroblasts and **(B)** keratinocytes were incubated for 24 h with conditioned medium from healthy controls or cells exposed to either a single or recurring dose of hydrogen peroxide. While there was no effect on fibroblast viability, there was a decrease in keratinocyte activity due to the calcium concentrations in the conditioned medium. Additionally, there was a marked decrease in keratinocyte viability after incubation with the conditioned medium from recurring dose group compared to the single dose group. Significance (p < 0.05) is represented with *. Bars represent mean ± SEM. Data was analyzed with one-way ANOVA test with Tukey’s *post hoc* test. n = 3 for all groups. Significance (p < 0.05) is represented with *. Bars represent mean ± SEM.

While senescent cells lose their ability to proliferate, we wanted to investigate their ability to affect the otherwise healthy surrounding cells. In response to CM from senescent groups, there was no change in fibroblast viability (Figure 6A). However, in keratinocytes there was a decrease in all groups exposed to conditioned medium (Figure 6B). Note that the calcium from the fibroblast CM reduces viability of keratinocytes. But the lowest keratinocyte viability was observed in the group exposed to CM from cells exposed to recurring stress when compared with the CM from the single-dose stressed group (p = 0.027).

### 3.7 Recurring oxidative stress increased IL-1β gene expression but that did not translate to increased protein secretion

**FIGURE 7:**
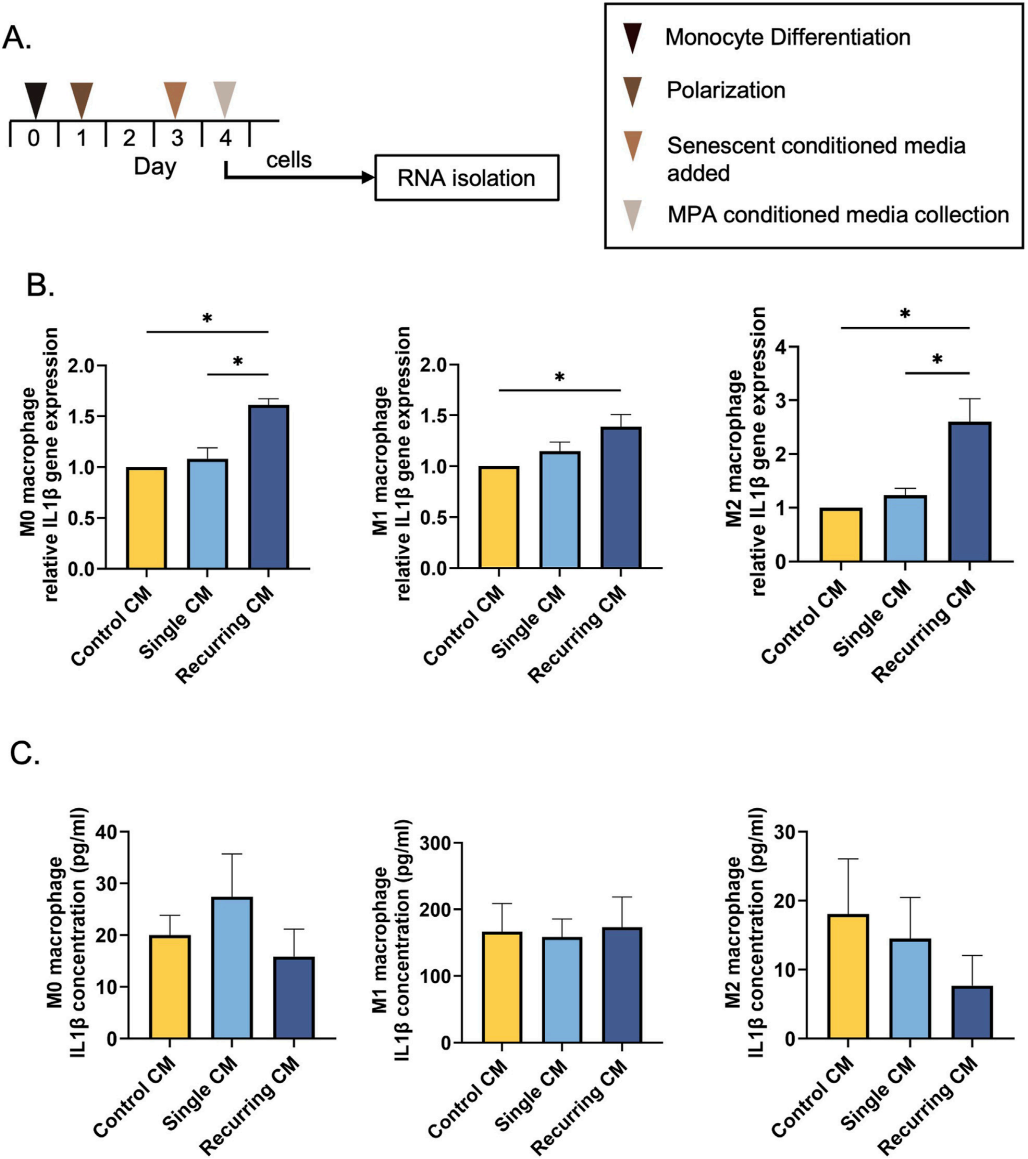
Recurring oxidative stress increases IL-1β gene expression. **(A)** THP-1 monocytes were differentiated into macrophages using 20 ng/mL PMA. Macrophages were either left unpolarized (M0) or polarized into an M1 or M2 phenotype. Lastly, macrophages were incubated with CM from senescent cells generated using a single or recurring H_2_O_2_ treatment. **(B)** IL-1β gene expression was investigated after incubation with control and senescent CM. There was an increase in IL-1β gene expression in all macrophage phenotypes after being treated with CM from the recurring H_2_O_2_ treatments. **(C)** The IL-1β protein concentration was also quantified but there were no differences in secretions per group. Data was analyzed with one-way ANOVA with Tukey’s *post hoc* test. n = 3 for all groups. Significance (p < 0.05) is represented with *. Bars represent mean ± SEM.

We next investigated whether the CM derived from fibroblasts exposed to different levels of oxidative stress would affect macrophage polarization (Figure 7A). When incubated with CM from untreated and oxidatively induced senescent fibroblasts, there was no surface marker changes observed in any group (Supplementary Material). There were also no changes in surface marker (*CD68, CD80, CD206*) gene expression or *CCL13* gene expression (Supplementary Material). However, we did observe a significant increase in IL-1β gene expression in M0, M1, and M2 macrophages when treated with CM from the recurring exposure group (Figure 7B). When IL-1β secretion concentrations were quantified after CM incubation, however, there was no significant differences between any groups (Figure 7C). A cytokine array was used to investigate the secretome of control, single stress, and recurring stress fibroblasts. Interestingly, compared to the control, there was a decrease in secretome diversity after senescence induction in both single and recurring oxidatively stressed groups (Supplementary Material).

## 4 Discussion

In this study, we wanted to determine the difference between two different methods of inducing senescence *in vitro*. Since oxidative stress is a key driver of skin stress, we compared senescent fibroblasts generated from a single dose of H_2_O_2_ to those generated from recurring exposure of H_2_O_2_. As the role of senescence and age is currently being dissected, this work shows that there is a correlation between increased exposure to oxidative stress and a more dysfunctional senescent phenotype. We observed these differences in measures of senescence in the cells themselves, as well as in the response of other cells found in skin to the conditioned media from these cells.

The fibroblasts exposed to oxidative stress had an increased cellular and nuclear area in response to both single and recurring oxidative stress. We observed that there was a severity-dependent response, however, with the cells that were stressed multiple times having a larger nucleic area and total cell area compared to untreated controls and fibroblasts that were only exposed once to oxidative stress. We also observed that while the cells ballooned in area, the N:C ratio between senescent groups were not statistically different, suggesting that both the cytoplasm and nucleus increase in tandem. Recently, researchers have shown that enlarged and flattened cells have diluted cytoplasmic proteins and are more prone to senescence, resulting in changes to cellular functions (Belhadj et al., 2023; Lanz et al., 2022; Neurohr et al., 2019). Additionally, we observed an increase in micronuclei in the fibroblasts that were treated with H_2_O_2_. Under stressed conditions, micronuclei arise from chromosome fragments that are erroneously left out of the nucleus and since cells recognize these micronuclei as foreign, they develop a pro-inflammatory secretory phenotype, commonly seen in senescent cells (Glück et al., 2017; Dou et al., 2017; Krupina, Goginashvili, and Cleveland, 2021).

Since SABGAL is one of the most widely used markers to determine senescence in cells, we wanted to investigate the differences in SABGAL expression between a single dose and recurring doses of H_2_O_2_. In addition to morphological changes, we observed an increase in SABGAL expression in our generated senescent cells compared to untreated controls. Furthermore, we observed that fibroblasts exposed to recurring oxidative stress had a higher population of senescent cells (79.0%) when compared to singly dosed cells and untreated controls and (40% and 14%, respectively). Clinically, SABGAL has been used to correlate senescence and pathological conditions. For example, when aortic tissues from healthy and atherosclerotic patents were examined for senescent cells, researchers observed that SABGAL positive endothelial cells overlapped with atherosclerotic plaques in aortic tissues (Vasile et al., 2001). Our work suggests that there can also be a link between severity of oxidative stress exposure and SABGAL expression.

Additionally, we investigated if normal cellular activity was disrupted due to senescence induction. We observed a decrease in cellular viability, decrease in Ki67 expression, and a massive reduction in colony formation capacity as a response to oxidative stress in both senescent groups. Furthermore, we went on to investigate cell cycle inhibitors *CDKN2A* and *CDKN1A*. We observed that cells that were recurringly stressed had increased expression of both *CDKN2A* and *CDKN1A,* while there was no difference between healthy and acutely stressed fibroblasts. Again, this suggests that there is a severity-dependent correlation between cell cycle arrest and oxidative stress. Previously, researchers have shown that endogenous oxidative stress can lead to an increase in replication stress which can lead to replication errors and tumor formation. As a protective mechanism the replication velocity decreases in response to oxidative stress (Wilhelm et al., 2016). In addition, researchers have shown that oxidative stress can lead to the accumulation of DNA damage (Ogrunc et al., 2014). As a result, we went on to investigate the effect of endogenous oxidative stress on DNA. Our data shows an increase in *γ*H2AX expression in both senescent groups with the single exposure group showing signs of recovery by day 5. This data characterizes both the DNA damage response and cessation of a healthy cell cycle in single and recurringly stressed fibroblasts. Taken together, our data shows that in response to recurring pulses of low oxidative stress, there is a purer population of damaged, non-proliferating cells.

Unfortunately, it is still unclear from the literature whether the more “severe” their phenotype becomes the more dysfunctional they are. While this works’ goal begins to answer this question, further research needs to be conducted to elucidate the relationship between changes in senescence, cellular size, abnormal cell cycles and dysfunctional cells. While the SABGAL staining demonstrates that there are higher percentages of senescent cells in our recurringly exposed cultures, the (normalized) RNA data for *CDKN2A* and *CDKN1A* also suggests that these cells are in a state of cell cycle arrest. Thus, our protocol utilizing recurring exposure to oxidative stress produces an overall more intense senescent culture due to individual cell changes as well as numbers of cells that become senescent.

While we have shown that lamin B1 expression decreases in response to oxidative stress, the implications between lamin B1 expression and cellular health are not clear. Researchers have observed that lamin B1 overexpression is correlated with a cell’s inability to repair damaged DNA, resulted in persistent DNA damage, and increased sensitivity to double-strand breaks (Barascu et al., 2012; Etourneaud et al., 2021). Conversely, researchers have also shown that lamin B1 loss can be correlated to DNA damage oncogenic activation, and replicative exhaustion, as seen in replicative-senescent cells (Freund et al., 2012; Wang et al., 2017; Saleh et al., 2022). While we have shown that there is a correlation between senescent markers and lamin B1 expression in our studies, more research needs to be done to parse out the relationship between senescence and lamin B1 expression.

Within the dermis, fibroblasts are the most prominent cells since they are responsible for synthesis and organization of proteins, such as collagen, which provide structural support of the skin. To investigate the role of senescence on collagen production in fibroblasts, we investigated the gene expression of collagen-I (*COL1A1*), collagen-III (*COL3A1*), and collagen-IV (*COL4A1*), which are all important proteins in promoting healthy skin architecture and skin wound healing (Mathew-Steiner et al., 2021). This work has shown that there was a marked decrease in collagen gene expression in both senescent groups, but that there was a senescence severity-dependent response specifically in collagen-I, where there was a significant decrease in the cells generated from recurring oxidative stress compared to the single dose group. The connection between age, senescence, and extracellular matrix have been extensively reviewed (Guvatova et al., 2022; Mavrogonatou et al., 2023). In brief, researchers have determined that there is a link between oxidative stress and ECM health. As aged cells have repeatedly been exposed to oxidative stress the senescent population increases. This then decreases collagen deposition and increases collagen fragmentation, resulting in thin and unsupportive ECM for fibroblasts (Tu and Quan, 2016; Fisher et al., 2009; He et al., 2014). Since collagen-I plays an important role in skin wound healing, this is consistent with the idea that recurring oxidative stress and the resulting senescent cells can hinder wound healing *in vivo*.

As a method of survival, senescent cells develop the machinery to escape apoptosis. Caspase-3 is an important apoptotic protein that, when activated, promotes a pathway to programmed cell death. We set out to investigate the homeostatic expression of caspase-3 in single and recurringly stressed fibroblasts. We determined that there were no differences in homeostatic caspase-3 expression in both senescent groups compared to healthy fibroblasts. To further investigate the ability of senescent fibroblasts to escape apoptosis, we incubated cells with the chemotherapeutic raptinal to promote apoptosis. Both groups of senescent fibroblasts were resistant to apoptosis and there was no expression of cleaved caspase-3, showing there was no activation of the apoptotic pathway. The state of literature on the role of caspase-3 in senescence is contradictory due to the multiple methods of senescence induction explored (Marcotte, Lacelle, and Wang, 2004; Ogata et al., 2021; Ohshima, 2004). Even though the direct relationship between caspase-3 and apoptosis in senescent cells is inconclusive, our data shows that oxidatively stressed fibroblasts can escape apoptosis and reside in the microenvironment.

Currently, senolytics are being investigated for the selective removal of senescent cells both *in vitro* and *in vivo* (Rad and Grillari, 2024; Takaya and Kishi, 2024; Zhu et al., 2016; Islam et al., 2023) with promising results. To further characterize our generated senescent cells, we investigated the effects of two senolytic treatments, Navitoclax and a D + Q cocktail. As expected, there was a decrease in SABGAL positive cells after treatment with Navitoclax, which works by blocking senescent cells ability to inhibit apoptosis and triggers cell death (Czabotar et al., 2014). We also observed that the recurringly stressed cells were more sensitive to lower concentrations of the treatment compared to the single stressed cells. By contrast, for D + Q we observed the same effect in both the single and recurringly stressed cells–a slight increase in SABGAL positive cells after treatment. Consistent with this observation, a temporary increase in SABGAL in response to D + Q has previously been observed in vascular smooth muscle cells attributed to cellular stress during treatment. (Gadecka et al., 2025).

Senescent cells have a distinct secretome that differs from healthy cells and the SASP is heavily dependent on the cell type and senescence induction method. Thus, we investigated the secretory changes of the resulting senescent cells and the effects they could have on healthy surrounding cells. After being treated with CM from recurringly stressed fibroblasts, there was a decrease in HUVEC tubule formation. Promoting healthy vasculature is critical in skin wound healing and for overall tissue health. This data suggests there is crosstalk between senescent fibroblasts and vascular cells; however, we have not identified any previous research reporting this, motivating further investigation of this novel finding. In fibroblasts, there was no effect of the SASP; yet, there was a decrease in keratinocyte viability in response to recurringly stressed CM compared to single dose group. This suggests that senescent fibroblasts in the dermis can release factors that reduce the health and function of keratinocytes in the epidermis, which in turn can affect tissue functionality and re-epithelialization–a critical component of wound healing.

Lastly, we wanted to investigate the role of SASPs on macrophages, which play a pivotal role in skin wound healing. When macrophages were incubated with the CM from recurringly stressed fibroblasts, there was an increase in *IL-1β* gene expression in all macrophage phenotypes compared to healthy fibroblasts. Further, in M0 and M2 macrophages, we observed a oxidative stress severity-dependent response in which cells treated with the recurring group CM also had increased *IL-1β* gene expression compared to CM from singly stressed fibroblasts. Despite this, there was no difference in IL-1β protein secretion in macrophages as measured by ELISA, alluding to the lack of translation of these genes. Researchers have shown that IL-1β is commonly increased in the SASP of senescent cells regardless of cell type, promoting their pro-inflammatory phenotype (Liu et al., 2021; Lupa et al., 2015; Nakamura et al., 2021; Victorelli et al., 2023). However, we did not observe an increase in secreted IL-1β from macrophages treated with either senescent SASPs. To further probe the secretome from the fibroblasts for pro-inflammatory cytokines, we investigated an additional 35 cytokines and observed an overall increase in pro-inflammatory SASP in the single oxidative stress group compared to the recurringly stressed group and control (Supplementary Material). Researchers have previously shown that the SASP from senescent cells acts as a chemoattractant to recruit immune cells for eventual clearance (Kale et al., 2020; Burton and Alexandra, 2018). Since the fibroblasts that were exposed to a single dose of hydrogen peroxide showed increased pro-inflammatory cytokine expression, we can assume that these cells have the potential to be cleared from the body. While we anticipated a higher immune response in the recurring group, there may be other aspects of their signaling that play a role in inflammation. Macrophages have been identified as key contributors to inflammaging, with senescent-associated macrophages (SAMs) increasing with age (Hall et al., 2016). While these SAMs were identified *in vivo* in response to the SASP, the exact elements of the SASP that triggered the macrophages is still under investigation (Hall et al., 2016). Here, we show that the induction method used yields different secretory profiles, but further studies are required to explore the total composition of the SASPs to elucidate the overall nature of SASP differences and the ultimate biological role *in vivo*. Lastly, we would like to acknowledge that there are many methods of generating macrophages from THP-1 monocytes and researchers are working to illuminate the correlations between differentiation and polarization methods and the resulting macrophage phenotype (Starr et al., 2018; Lund et al., 2016). While we have not observed differences in macrophage surface marker expression in response to our generated SASP, other induction methods may generate different results.

Our research has shown that in most circumstances we tested, the secretome of senescent cells generated using one dose of H_2_O_2_ behaved similarly to healthy fibroblasts. This suggests that as fibroblasts are introduced to long-term, recurring stress, they become more indicative of aged, unhealthy cells. While we incubated healthy cells with CM from senescent fibroblasts, this method is not indicative of the complete complexity of skin and the multiple cell types within. More complex systems should be implemented to investigate the complex relationship between senescent fibroblasts and surrounding cell types *in vivo*. Additionally, while we have focused on healthy fibroblasts and skin wound healing in this study, researchers have shown that oncogenic induction also causes an increase in cellular oxidation (Ogrunc et al., 2014). This stress can be correlated with DNA damage and senescence and fibroblasts are abundant in many tumors suggesting that this work also has implications in cancer biology.

## 5 Conclusion

While there are many ways of inducing senescence *in vitro* (H_2_O_2_ mediated oxidative stress, oncogenic induction, telomere shortening via replicative exhaustion, etc.), this work shows that that the exposure levels of oxidative stress dictates the resulting cellular and secretory characteristics of the generated cells. In particular, the fibroblasts that received a single 500 µM dose of H_2_O_2_ behaved similarly to the healthy control, whereas they differed from the cells that were exposed to recurring stress. We have also observed that the severity of oxidative stress correlates with the response that healthy cells have to the secretome generated by the senescent cells. This relatively short method of inducing senescence in cells, which does not require passaging or extended treatments, can now be used to assess and mimic aging and senescence within *in vitro* studies and can be readily adapted for other cell types and applications.

## Data Availability

The raw data supporting the conclusions of this article will be made available by the authors, without undue reservation.

## References

[B1] Barascu A. Le Chalony C. Pennarun G. Genet D. Imam N. Lopez B. (2012). Oxidative stress induces an ATM-independent senescence pathway through P38 MAPK-mediated lamin B1 accumulation. EMBO J. 31 (5), 1080–1094. 10.1038/emboj.2011.492 22246186 PMC3297999

[B2] Barber L. A. Spandau D. F. Rathman S. C. Murphy R. C. Johnson C. A. Kelley S. W. (1998). Expression of the platelet-activating factor receptor results in enhanced ultraviolet B radiation-induced apoptosis in a human epidermal cell line. J. Biol. Chem. 273 (30), 18891–18897. 10.1074/jbc.273.30.18891 9668065

[B3] Basisty N. Kale A. Jeon Ok H. Kuehnemann C. Payne T. Rao C. (2020). “A proteomic atlas of senescence-associated secretomes for aging biomarker development,” PLOS Biol., 18. 10.1371/journal.pbio.3000599 PMC6964821 31945054

[B4] Belhadj J. Surina S. Hengstschläger M. Lomakin A. J. (2023). Form follows function: nuclear morphology as a quantifiable predictor of cellular senescence. Aging Cell 22 (12), e14012. 10.1111/acel.14012 37845808 PMC10726876

[B5] Birch J. Gil J. (2020). Senescence and the SASP: many therapeutic avenues. Genes and Dev. 34 (23–24), 1565–1576. 10.1101/gad.343129.120 33262144 PMC7706700

[B6] Burnaevskiy N. Oshima J. Mendenhall A. R. (2022). Oxidative stress induced senescence gives rise to transcriptionally distinct physiological states. 10.1101/2022.05.18.492555

[B7] Burton D. G. A. Alexandra S. (2018). Cellular senescence: immunosurveillance and future immunotherapy. Ageing Res. Rev. 43 (May), 17–25. 10.1016/j.arr.2018.02.001 29427795

[B8] Chen Q. Ames B. N. (1994). Senescence-like growth arrest induced by hydrogen peroxide in human diploid fibroblast F65 cells. Proc. Natl. Acad. Sci. 91 (10), 4130–4134. 10.1073/pnas.91.10.4130 8183882 PMC43738

[B9] Coppé J.-P. Desprez P.-Y. Krtolica A. Campisi J. (2010). The senescence-associated secretory phenotype: the dark side of tumor suppression. Annu. Rev. Pathology 5, 99–118. 10.1146/annurev-pathol-121808-102144 PMC4166495 20078217

[B10] Czabotar P. E. Lessene G. Strasser A. Adams J. M. (2014). Control of apoptosis by the BCL-2 protein family: implications for physiology and therapy. Nat. Rev. Mol. Cell Biol. 15 (1), 49–63. 10.1038/nrm3722 24355989

[B11] Demaria M. Ohtani N. Youssef S. A. Rodier F. Toussaint W. Mitchell J. R. (2014). An essential role for senescent cells in optimal wound healing through secretion of PDGF-AA. Dev. Cell 31 (6), 722–733. 10.1016/j.devcel.2014.11.012 25499914 PMC4349629

[B12] Deryabin P. I. Shatrova A. N. Borodkina A. V. (2021). Apoptosis resistance of senescent cells is an intrinsic barrier for senolysis induced by cardiac glycosides. Cell. Mol. Life Sci. 78 (23), 7757–7776. 10.1007/s00018-021-03980-x 34714358 PMC8629786

[B13] Dou Z. Ghosh K. Vizioli M. G. Zhu J. Sen P. Wangensteen K. J. (2017). Cytoplasmic chromatin triggers inflammation in senescence and cancer. Nature 550 (7676), 402–406. 10.1038/nature24050 28976970 PMC5850938

[B14] Etourneaud L. Moussa A. Rass E. Genet D. Willaume S. Chabance-Okumura C. (2021). Lamin B1 sequesters 53BP1 to control its recruitment to DNA damage. Sci. Adv. 7 (35), eabb3799. 10.1126/sciadv.abb3799 34452908 PMC8397269

[B15] Facchin F. Bianconi E. Romano M. Impellizzeri A. Alviano F. Maioli M. (2018). Comparison of oxidative stress effects on senescence patterning of human adult and perinatal tissue-derived stem cells in short and long-term cultures. Int. J. Med. Sci. 15 (13), 1486–1501. 10.7150/ijms.27181 30443170 PMC6216057

[B16] Fagagna F. d’A.Di Reaper P. M. Clay-Farrace L. Fiegler H. Carr P. Von Zglinicki T. (2003). A DNA damage checkpoint response in telomere-initiated senescence. Nature 426 (6963), 194–198. 10.1038/nature02118 14608368

[B17] Feng T. Meng J. Kou S. Jiang Z. Huang X. Lu Z. (2019). CCN1-Induced cellular senescence promotes heart regeneration. Circulation 139 (21), 2495–2498. 10.1161/CIRCULATIONAHA.119.039530 31107624

[B18] Fisher G. J. Quan T. Purohit T. Shao Y. Cho M. K. He T. (2009). Collagen fragmentation promotes oxidative stress and elevates matrix metalloproteinase-1 in fibroblasts in aged human skin. Am. J. Pathology 174 (1), 101–114. 10.2353/ajpath.2009.080599 PMC2631323 19116368

[B19] Freund A. Laberge R.-M. Demaria M. Campisi J. (2012). Lamin B1 loss is a senescence-associated biomarker. Mol. Biol. Cell 23 (11), 2066–2075. 10.1091/mbc.E11-10-0884 22496421 PMC3364172

[B20] Gadecka A. Nowak N. Bulanda E. Janiszewska D. Dudkowska M. Sikora E. (2025). The senolytic cocktail, Dasatinib and Quercetin, impacts the chromatin structure of both young and senescent vascular smooth muscle cells. GeroScience, January. 10.1007/s11357-024-01504-6 PMC12181558 39828770

[B21] Gerasymchuk M. Robinson G. I. Kovalchuk O. Kovalchuk I. (2022). Modeling of the senescence-associated phenotype in human skin fibroblasts. Int. J. Mol. Sci. 23 (13), 7124. 10.3390/ijms23137124 35806127 PMC9266450

[B22] Glück S. Guey B. Gulen M. F. Wolter K. Kang T.-W. Schmacke N. A. (2017). Innate immune sensing of cytosolic chromatin fragments through cGAS promotes senescence. Nat. Cell Biol. 19 (9), 1061–1070. 10.1038/ncb3586 28759028 PMC5826565

[B23] Grosse L. Wagner N. Emelyanov A. Molina C. Lacas-Gervais S. Wagner K.-D. (2020). Defined p16High senescent cell types are indispensable for mouse healthspan. Cell Metab. 32 (1), 87–99.e6. 10.1016/j.cmet.2020.05.002 32485135

[B24] Guvatova Z. G. Borisov P. V. Alekseev A. A. Moskalev A. A. (2022). Age-related changes in extracellular matrix. Biochem. Mosc. 87 (12–13), 1535–1551. 10.1134/S0006297922120112 36717445

[B25] Hall B. M. Balan V. Gleiberman A. S. Strom E. Krasnov P. Virtuoso L. P. (2016). Aging of mice is associated with P16(ink4a)- and β-galactosidase-positive macrophage accumulation that can Be induced in young mice by senescent cells. Aging 8 (7), 1294–1315. 10.18632/aging.100991 27391570 PMC4993332

[B26] Hayflick L. Moorhead P. S. (1961). The serial cultivation of human diploid cell strains. Exp. Cell Res. 25 (3), 585–621. 10.1016/0014-4827(61)90192-6 13905658

[B27] He T. Quan T. Shao Y. Voorhees J. J. Fisher G. J. (2014). Oxidative exposure impairs TGF-β pathway via reduction of type II receptor and SMAD3 in human skin fibroblasts. AGE 36 (3), 9623. 10.1007/s11357-014-9623-6 24550076 PMC4082581

[B28] Hu Li Li H. Zi M. Wen Li Liu J. Yang Y. (2022). Why senescent cells are resistant to apoptosis: an insight for senolytic development. Front. Cell Dev. Biol. 10 (February), 822816. 10.3389/fcell.2022.822816 35252191 PMC8890612

[B29] Islam Md T. Tuday E. Allen S. Kim J. Trott D. W. Holland W. L. (2023). Senolytic drugs, Dasatinib and Quercetin, attenuate adipose tissue inflammation, and ameliorate metabolic function in old age. Aging Cell 22 (2), e13767. 10.1111/acel.13767 36637079 PMC9924942

[B30] Jeon Ok H. Kim C. Laberge R.-M. Demaria M. Rathod S. Vasserot A. P. (2017). Local clearance of senescent cells attenuates the development of post-traumatic osteoarthritis and creates a pro-regenerative environment. Nat. Med. 23 (6), 775–781. 10.1038/nm.4324 28436958 PMC5785239

[B31] Kale A. Sharma A. Stolzing A. Desprez P.-Y. Campisi J. (2020). Role of immune cells in the removal of deleterious senescent cells. Immun. and Ageing 17 (1), 16. 10.1186/s12979-020-00187-9 PMC7271494 32518575

[B32] Kiyoshima T. Enoki N. Kobayashi I. Sakai T. Nagata K. Wada H. (2012). Oxidative stress caused by a low concentration of hydrogen peroxide induces senescence-like changes in mouse gingival fibroblasts. Int. J. Mol. Med. 30 (5), 1007–1012. 10.3892/ijmm.2012.1102 22922974 PMC3573718

[B33] Krupina K. Alexander G. Cleveland D. W. (2021). Causes and consequences of micronuclei. Curr. Opin. Cell Biol. 70 (June), 91–99. 10.1016/j.ceb.2021.01.004 33610905 PMC8119331

[B34] Laliberté C. Bossé B. Bourdeau V. Prieto L. I. Perron-Deshaies G. Vuong-Robillard N. (2024). Senescent macrophages release inflammatory cytokines and RNA-loaded extracellular vesicles to circumvent fibroblast senescence. Biomedicines 12 (5), 1089. 10.3390/biomedicines12051089 38791051 PMC11118806

[B35] Lanz M. C. Zatulovskiy E. Swaffer M. P. Zhang L. Ilerten I. Zhang S. (2022). Increasing cell size remodels the Proteome and promotes senescence. Mol. Cell 82 (17), 3255–3269.e8. 10.1016/j.molcel.2022.07.017 35987199 PMC9444988

[B36] Liton P. B. Challa P. Stinnett S. Luna C. Epstein D. L. Gonzalez P. (2005). Cellular senescence in the glaucomatous outflow pathway. Exp. Gerontol. 40 (8–9), 745–748. 10.1016/j.exger.2005.06.005 16051457 PMC3152456

[B37] Liu W. Brodsky A. S. Feng M. Liu Y. Ding J. Jayasuriya C. T. (2021). Senescent tissue-resident mesenchymal stromal cells are an internal source of inflammation in human osteoarthritic cartilage. Front. Cell Dev. Biol. 9 (September), 725071. 10.3389/fcell.2021.725071 34552931 PMC8450518

[B38] Low E. Alimohammadiha G. Smith L. A. Costello L. F. Przyborski S. A. Von Zglinicki T. (2021). How good is the evidence that cellular senescence causes skin ageing? Ageing Res. Rev. 71 (November), 101456. 10.1016/j.arr.2021.101456 34487917 PMC8524668

[B39] Lund M. E. Joyce To O’Brien B. A. Donnelly S. (2016). The choice of phorbol 12-myristate 13-acetate differentiation protocol influences the response of THP-1 macrophages to a pro-inflammatory stimulus. J. Immunol. Methods 430 (March), 64–70. 10.1016/j.jim.2016.01.012 26826276

[B40] Lupa W. M. D. Kalfalah F. Safferling K. Boukamp P. Poschmann G. (2015). Characterization of skin aging–associated secreted proteins (SAASP) produced by dermal fibroblasts isolated from intrinsically aged human skin. J. Investigative Dermatology 135 (8), 1954–1968. 10.1038/jid.2015.120 25815425

[B41] Marcotte R. Lacelle C. Wang E. (2004). Senescent fibroblasts resist apoptosis by downregulating caspase-3. Mech. Ageing Dev. 125 (10–11), 777–783. 10.1016/j.mad.2004.07.007 15541772

[B42] Mathew-Steiner S. S. Roy S. Sen C. K. (2021). Collagen in wound healing. Bioengineering 8 (5), 63. 10.3390/bioengineering8050063 34064689 PMC8151502

[B43] Mavrogonatou E. Papadopoulou A. Pratsinis H. Kletsas D. (2023). Senescence-associated alterations in the extracellular matrix: deciphering their role in the regulation of cellular function. Am. J. Physiology-Cell Physiology 325 (3), C633–C647. 10.1152/ajpcell.00178.2023 37486063

[B44] Muñoz-Espín D. Cañamero M. Maraver A. Gómez-López G. Contreras J. Murillo-Cuesta S. (2013). Programmed cell senescence during mammalian embryonic development. Cell 155 (5), 1104–1118. 10.1016/j.cell.2013.10.019 24238962

[B45] Nakamura Y. Aihara R. Iwata H. Kuwayama T. Shirasuna K. (2021). IL1B triggers inflammatory cytokine production in bovine oviduct epithelial cells and induces neutrophil accumulation via CCL2. Am. J. Reproductive Immunol. 85 (5), e13365. 10.1111/aji.13365 33099841

[B46] Neurohr G. E. Terry R. L. Lengefeld J. Bonney M. Brittingham G. P. Moretto F. (2019). Excessive cell growth causes cytoplasm dilution and contributes to senescence. Cell 176 (5), 1083–1097.e18. 10.1016/j.cell.2019.01.018 30739799 PMC6386581

[B47] Ogata Y. Yamada T. Hasegawa S. Sanada A. Iwata Y. Arima M. (2021). SASP‐induced macrophage dysfunction may contribute to accelerated senescent fibroblast accumulation in the dermis. Exp. Dermatol. 30 (1), 84–91. 10.1111/exd.14205 33010063

[B48] Ogrunc M. Di Micco R. Liontos M. Bombardelli L. Mione M. Fumagalli M. (2014). Oncogene-induced reactive oxygen species fuel hyperproliferation and DNA damage response activation. Cell Death Differ. 21 (6), 998–1012. 10.1038/cdd.2014.16 24583638 PMC4013514

[B49] Ohshima S. (2004). Apoptosis in stress-induced and spontaneously senescent human fibroblasts. Biochem. Biophysical Res. Commun. 324 (1), 241–246. 10.1016/j.bbrc.2004.09.044 15465009

[B50] Palchaudhuri R. Lambrecht M. J. Botham R. C. Partlow K. C. van Ham T. J. Putt K. S. (2015). A small molecule that induces intrinsic pathway apoptosis with unparalleled speed. Cell Rep. 13 (9), 2027–2036. 10.1016/j.celrep.2015.10.042 26655912 PMC4683402

[B51] Purcu U. Korkmaz A. Gunalp S. Helvaci D. G. Erdal Y. Dogan Y. (2022). “Effect of stimulation time on the expression of human macrophage polarization markers, PLOS ONE, 17. 10.1371/journal.pone.0265196 PMC8920204 35286356

[B52] Rad A. N. Grillari J. (2024). Current senolytics: mode of action, efficacy and limitations, and their future. Mech. Ageing Dev. 217 (February), 111888. 10.1016/j.mad.2023.111888 38040344

[B53] Rebehn L. Khalaji S. KleinJan F. Kleemann A. Port F. Paul P. (2023). The weakness of senescent dermal fibroblasts. Proc. Natl. Acad. Sci. 120 (34), e2301880120. 10.1073/pnas.2301880120 37579160 PMC10450655

[B54] Saleh T. Alhesa A. El-Sadoni M. Shahin N. A. Alsharaiah E. Shboul S.Al (2022). The expression of the senescence-associated biomarker lamin B1 in human breast cancer. Diagnostics 12 (3), 609. 10.3390/diagnostics12030609 35328162 PMC8947068

[B55] Santarella F. Correa do Amaral R. J. F. Lemoine M. Kelly D. Cavanagh B. Marinkovic M. (2022). Personalized scaffolds for diabetic foot ulcer healing using extracellular matrix from induced pluripotent stem‐reprogrammed patient cells. Adv. NanoBiomed Res. 2 (10), 2200052. 10.1002/anbr.202200052 36532145 PMC9757804

[B56] Santarella F. Sridharan R. Marinkovic M. Amaral R. J. F. C.Do Cavanagh B. Smith A. (2020). Scaffolds functionalized with matrix from induced pluripotent stem cell fibroblasts for diabetic wound healing. Adv. Healthc. Mater. 9 (16), 2000307. 10.1002/adhm.202000307 32597577

[B57] Schloesser D. Lindenthal L. Sauer J. Chung K.-J. Chavakis T. Griesser E. (2023). Senescent cells suppress macrophage-mediated corpse removal via upregulation of the CD47-QPCT/L Axis. J. Cell Biol. 222 (2), e202207097. 10.1083/jcb.202207097 36459066 PMC9723804

[B58] Serrano M. Lin A. W. McCurrach M. E. Beach D. Lowe S. W. (1997). Oncogenic ras provokes premature cell senescence associated with accumulation of P53 and p16INK4a. Cell 88 (5), 593–602. 10.1016/S0092-8674(00)81902-9 9054499

[B59] Sivoňová M. Tatarková Z. Ďuračková Z. Dobrota D. Lehotský J. Matáková T. (2007). Relationship between antioxidant potential and oxidative damage to lipids, proteins and DNA in aged rats. Physiological Res. 56, 757–764. 10.33549/physiolres.931094 17087608

[B60] Sone H. Kagawa Y. (2005). Pancreatic beta cell senescence contributes to the pathogenesis of type 2 diabetes in high-fat diet-induced diabetic mice. Diabetologia 48 (1), 58–67. 10.1007/s00125-004-1605-2 15624098

[B61] Sridharan R. Ryan E. J. Kearney C. J. Kelly D. J. O’Brien F. J. (2019). Macrophage polarization in response to collagen scaffold stiffness is dependent on cross-linking agent used to modulate the stiffness. ACS Biomaterials Sci. and Eng. 5 (2), 544–552. 10.1021/acsbiomaterials.8b00910 33405818

[B62] Starr T. Bauler T. J. Malik-Kale P. Steele-Mortimer O. (2018). “The phorbol 12-myristate-13-acetate differentiation protocol is critical to the interaction of THP-1 macrophages with Salmonella typhimurium, 13. 10.1371/journal.pone.0193601 PMC5851575 29538403

[B63] Stöckl P. Hütter E. Werner Z. Jansen-Dürr P. (2006). Sustained inhibition of oxidative phosphorylation impairs cell proliferation and induces premature senescence in human fibroblasts. Exp. Gerontol. 41 (7), 674–682. 10.1016/j.exger.2006.04.009 16713693

[B64] Storer M. Alba M. Robert-Moreno A. Pecoraro M. Carmen Ortells M. Di Giacomo V. (2013). Senescence is a developmental mechanism that contributes to embryonic growth and patterning. Cell 155 (5), 1119–1130. 10.1016/j.cell.2013.10.041 24238961

[B65] Takaya K. Kishi K. (2024). Combined Dasatinib and Quercetin treatment contributes to skin rejuvenation through selective elimination of senescent cells *in vitro* and *in vivo* . Biogerontology 25 (4), 691–704. 10.1007/s10522-024-10103-z 38619669

[B66] Terlecki-Zaniewicz L. Vera P. Reddy Bobbili M. Lämmermann I. Perrotta I. Grillenberger T. (2019). Extracellular vesicles in human skin: cross-talk from senescent fibroblasts to keratinocytes by miRNAs. J. Investigative Dermatology 139 (12), 2425–2436.e5. 10.1016/j.jid.2019.05.015 31220456

[B67] Tu Y. Quan T. (2016). Oxidative stress and human skin connective tissue aging. Cosmetics 3 (3), 28. 10.3390/cosmetics3030028

[B68] Vasile E. Tomita Y. Brown L. F. Kocher O. Dvorak H. F. (2001). Differential expression of thymosin Β‐10 by early passage and senescent vascular endothelium is modulated by VPF/VEGF: evidence for senescent endothelial cells *in vivo* at sites of atherosclerosis. FASEB J. 15 (2), 458–466. 10.1096/fj.00-0051com 11156961

[B69] Vernon M. Wilski N. A. Kotas D. Cai W. Pomante D. Tiago M. (2022). Raptinal induces gasdermin E–dependent pyroptosis in naïve and therapy-resistant melanoma. Mol. Cancer Res. 20 (12), 1811–1821. 10.1158/1541-7786.mcr-22-0040 36044013 PMC9722513

[B70] Victorelli S. Hanna S. Chapman J. Martini H. Vizioli M. G. Riley J. S. (2023). Apoptotic stress causes mtDNA release during senescence and drives the SASP. Nature 622 (7983), 627–636. 10.1038/s41586-023-06621-4 37821702 PMC10584674

[B71] Wallis R. Milligan D. Hughes B. Mizen H. López-Domínguez J. A. Eduputa U. (2022). Senescence-associated morphological profiles (SAMPs): an image-based phenotypic profiling method for evaluating the inter and intra model heterogeneity of senescence. Aging 14 (10), 4220–4246. 10.18632/aging.204072 35580013 PMC9186762

[B72] Wang A. S. Ong P. F. Chojnowski A. Clavel C. Dreesen O. (2017). Loss of lamin B1 is a biomarker to quantify cellular senescence in photoaged skin. Sci. Rep. 7 (1), 15678. 10.1038/s41598-017-15901-9 29142250 PMC5688158

[B74] Wang T.-W. Johmura Y. Suzuki N. Omori S. Migita T. Yamaguchi K. (2022). Blocking PD-L1–PD-1 improves senescence surveillance and ageing phenotypes. Nature 611 (7935), 358–364. 10.1038/s41586-022-05388-4 36323784

[B75] Watson R. E. B. Gibbs N. K. Griffiths C. E. M. Sherratt M. J. (2014). Damage to skin extracellular matrix induced by UV exposure. Antioxidants and Redox Signal. 21 (7), 1063–1077. 10.1089/ars.2013.5653 24124905

[B76] Wiley C. D. Velarde M. C. Lecot P. Liu Su Sarnoski E. A. Freund A. (2016). Mitochondrial dysfunction induces senescence with a distinct secretory phenotype. Cell Metab. 23 (2), 303–314. 10.1016/j.cmet.2015.11.011 26686024 PMC4749409

[B77] Wilhelm T. Ragu S. Magdalou I. Machon C. Dardillac E. Técher H. (2016). “Slow replication fork velocity of homologous recombination-defective cells results from endogenous oxidative stress,” PLOS Genet., 12. 10.1371/journal.pgen.1006007 PMC4852921 27135742

[B78] Wlaschek M. Maity P. Makrantonaki E. Scharffetter-Kochanek K. (2021). Connective tissue and fibroblast senescence in skin aging. J. Investigative Dermatology 141 (4), 985–992. 10.1016/j.jid.2020.11.010 33563466

[B79] Xu M. Su X. Xiao X. Yu H. Li X. Keating A. (2021). Hydrogen peroxide-induced senescence reduces the wound healing-promoting effects of mesenchymal stem cell-derived exosomes partially via miR-146a. Aging Dis. 12 (1), 102–115. 10.14336/AD.2020.0624 33532131 PMC7801275

[B80] Zhu Yi Tchkonia T. Fuhrmann‐Stroissnigg H. Dai H. M. Ling Y. Y. Stout M. B. (2016). Identification of a novel senolytic agent, Navitoclax, targeting the bcl‐2 family of anti‐apoptotic factors. Aging Cell 15 (3), 428–435. 10.1111/acel.12445 26711051 PMC4854923

[B81] Zonari A. Brace L. E. Al-Katib K. Porto W. F. Foyt D. Guiang M. (2023). Senotherapeutic peptide treatment reduces biological age and senescence burden in human skin models. Npj Aging 9 (1), 10. 10.1038/s41514-023-00109-1 37217561 PMC10203313

