# Supplementary material for: Human dermal fibroblast senescence in response to single and recurring oxidative stress
Source: Front Aging. 2025 Mar 28;6:1504977. doi: 10.3389/fragi.2025.1504977 (PMC11985536; doi:10.3389/fragi.2025.1504977)
Supplement: Supplementary file 1 [file DataSheet1.docx]

Supplementary Material


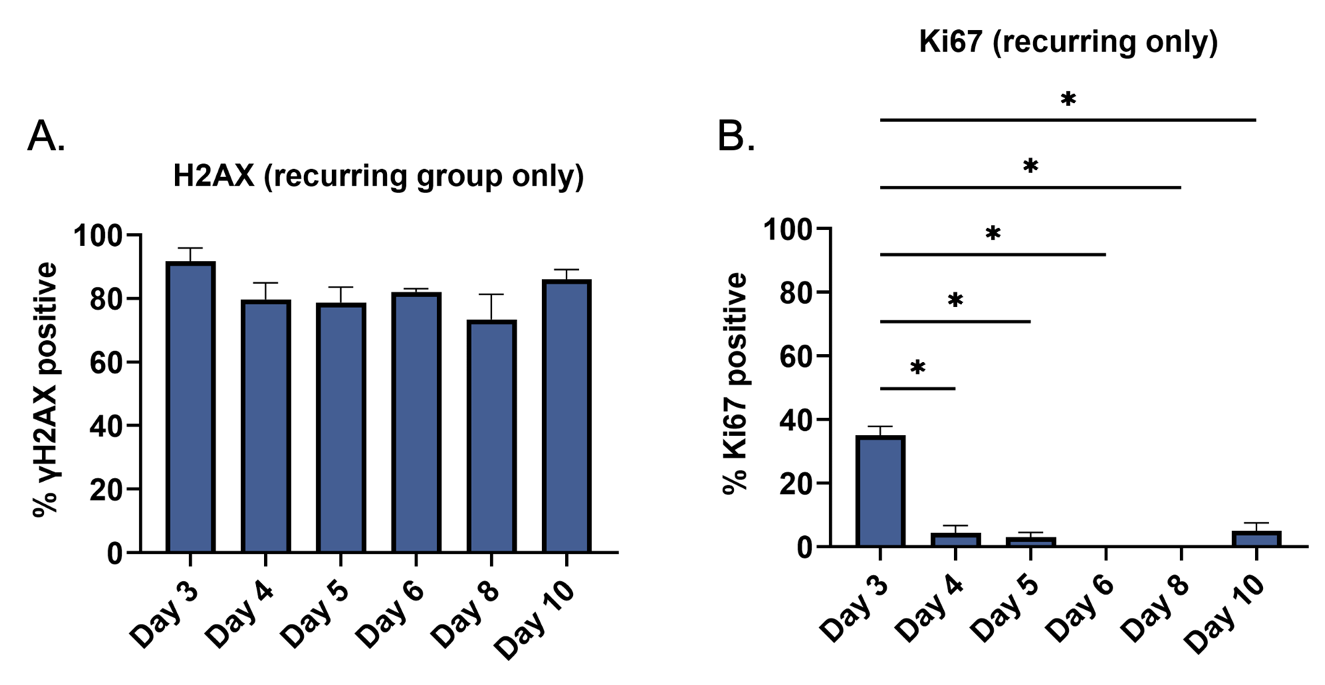


Supplementary Figure 1: Recurring oxidative stress results in sustained DNA damage and ceased proliferation. Senescence was induced in fibroblasts and DNA damage marker (𝛾H2AX) and proliferation marker (Ki67) were investigated. On days 3, 4, 5, 6, 8, and 10, the cells that were exposed to recurring oxidative stress were fixed and stained.


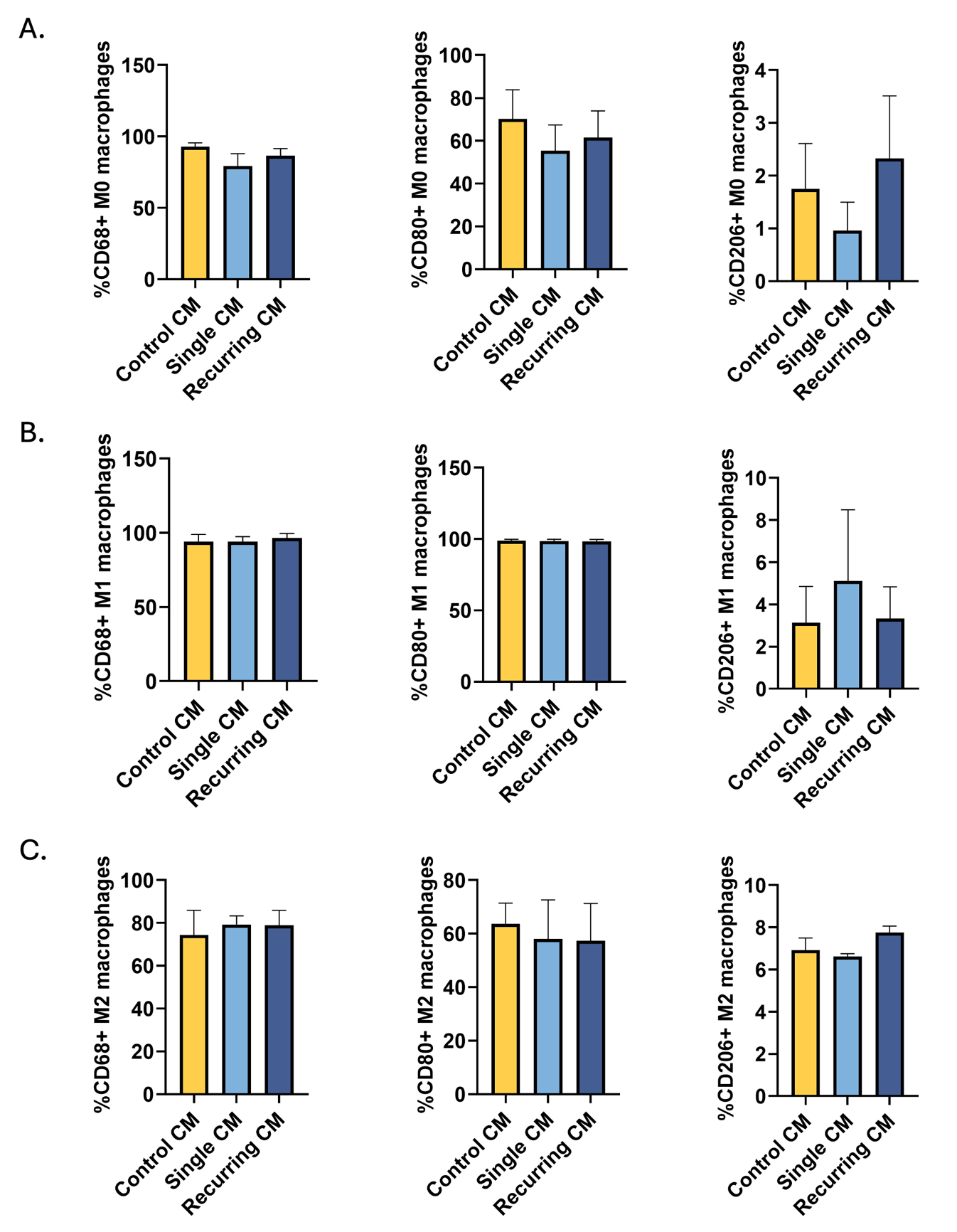


Supplementary Figure 2: There were no changes in macrophage surface marker expression in response to fibroblast conditioned media. (A) M0, (B) M1, and (C) M2 macrophage surface markers in response to healthy or senescent fibroblast conditioned media. Data was analyzed with one-way ANOVA (Tukey’s post-hoc test). N=3 for all groups. Bars represent mean ± standard error mean (SEM).


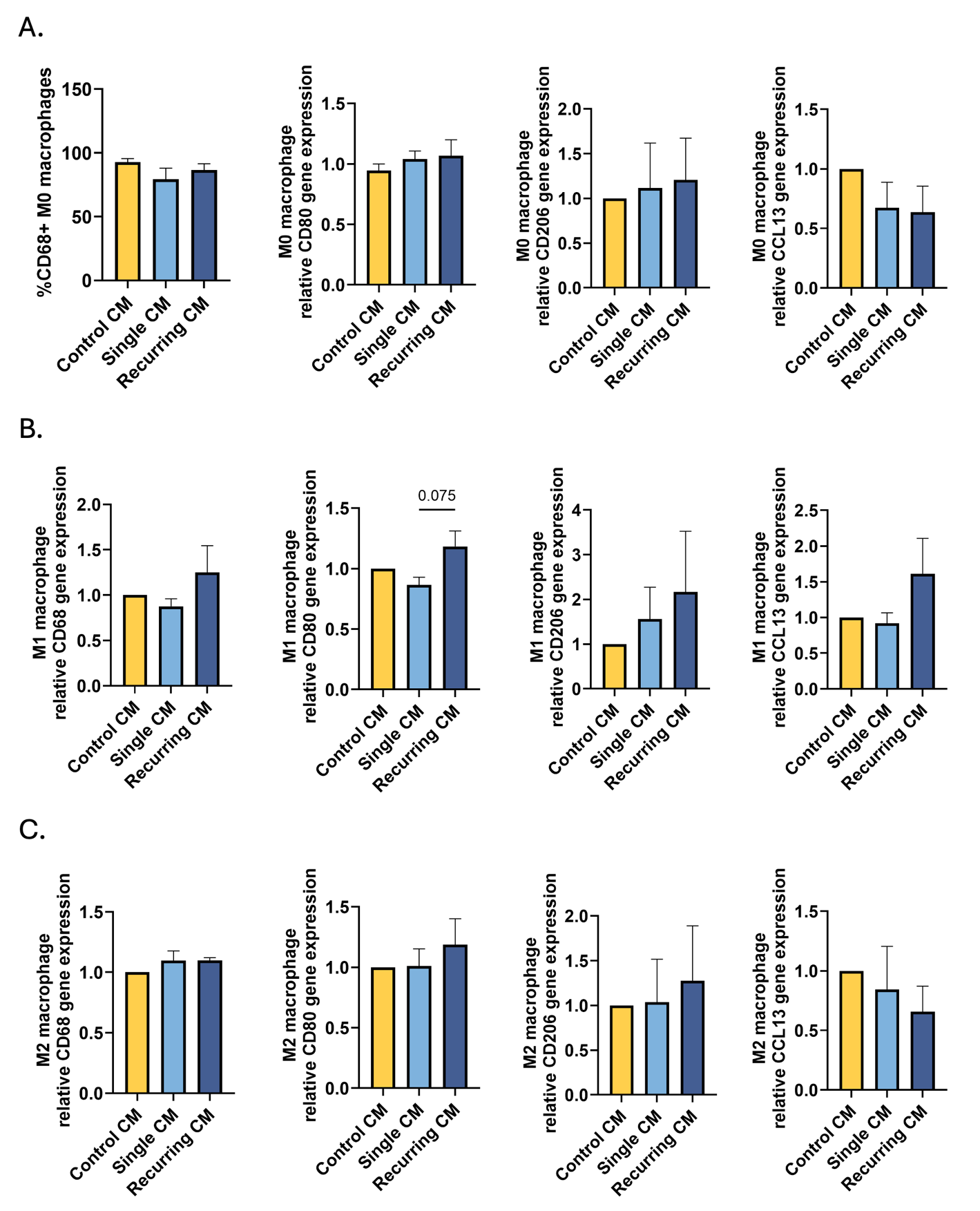


Supplementary Figure 3: There were no changes in macrophage gene expression in response to fibroblast conditioned media. (A) M0, (B) M1, and (C) M2 macrophage markers in response to healthy or senescent fibroblast conditioned media. Data was analyzed with one-way ANOVA (Tukey’s post-hoc test). N=3 for all groups. Bars represent mean ± standard error mean (SEM).


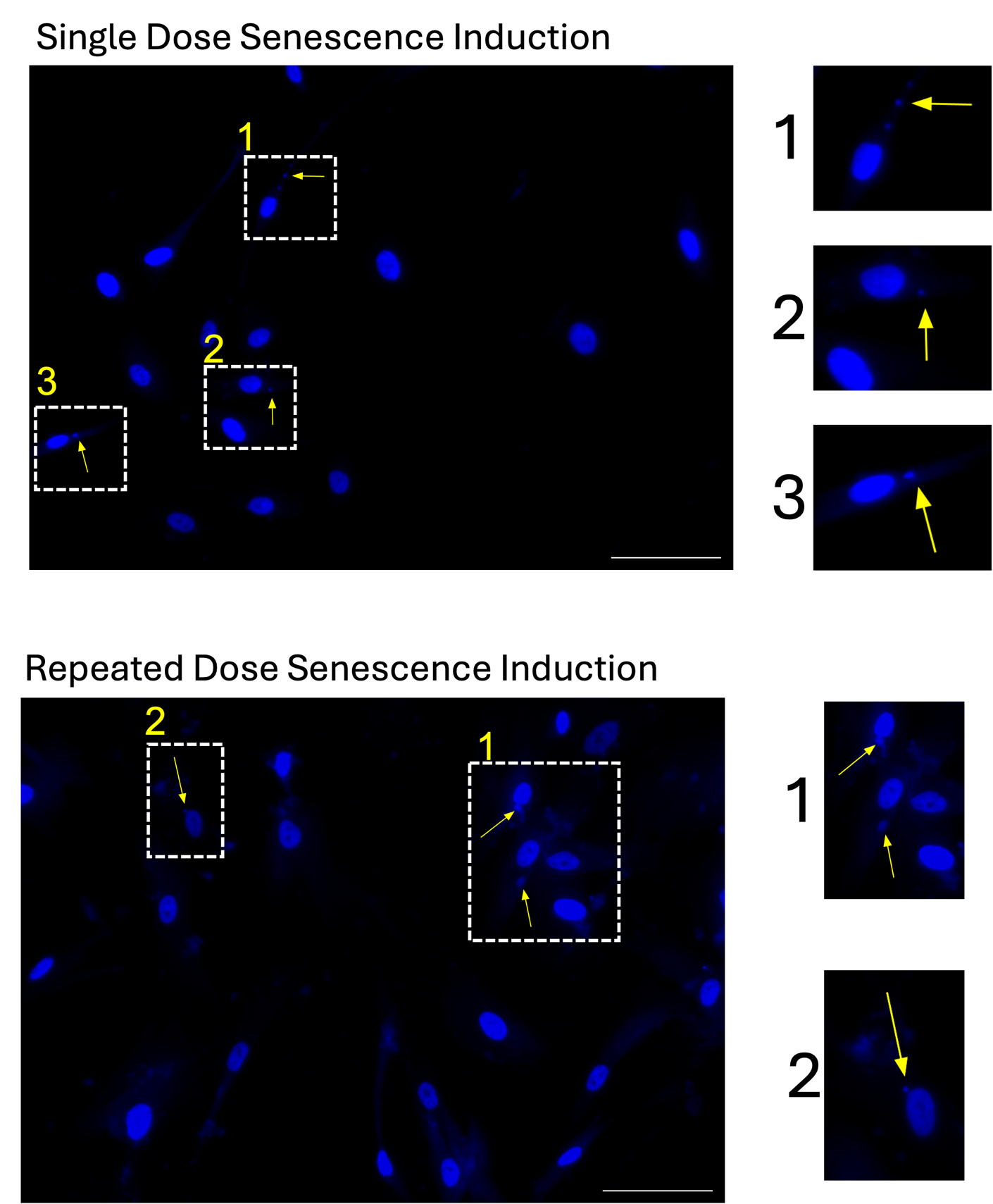


Supplementary Figure 4: Oxidative stress results in micronuclei formation.


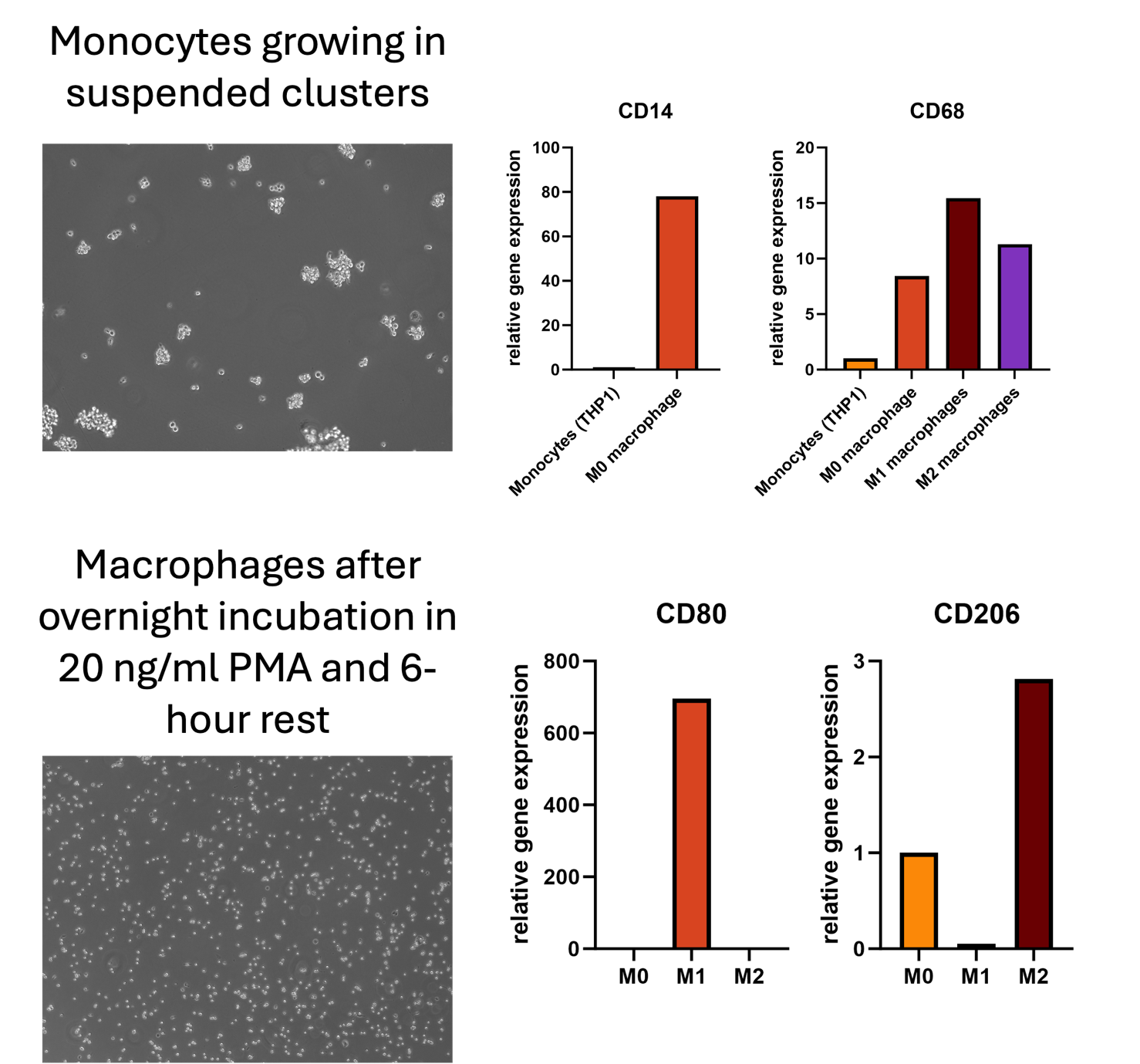


Supplementary Figure 5: Generation of macrophages from THP-1 monocytes. We incubated THP-1 cells with 20 ng/ml PMA overnight to promote differentiation to macrophages. The cells were then allowed to rest for 6 hours in PMA-free medium and polarized into M1 (LPS + IFN-γ) and M2 (IL-4 + IL-13) macrophages. The cells were then characterized to ensure that the method employed generated the expected phenotype.


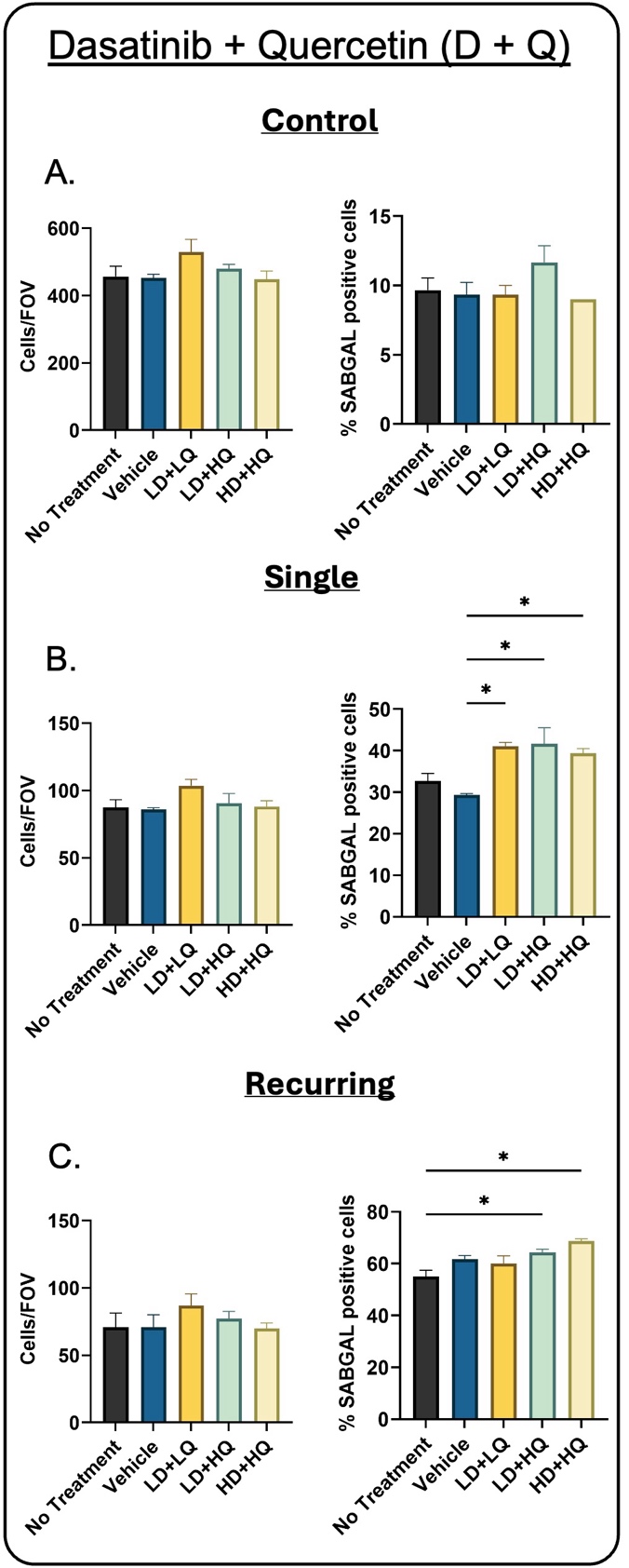


Supplemental Figure 6: D+Q cocktail increases SABGAL expression in senescent fibroblasts. (A) Healthy, (B) Single oxidatively stressed, and (C) recurring oxidatively stressed fibroblasts were treated with a Dasatinib and Quercetin (D+Q) cocktail at 3 varying low (L) and high (H) concentrations (LD= 100 nM, LQ = 10 µM, HD = 200 nM, HQ = 20 µM). In both the single and recurringly stressed groups, there was mostly an increase in SABGAL positive cells after treatment with the D+Q cocktail. Data was analyzed using one-way ANOVA test (Tukey’s post-hoc test). n = 3 for all groups. Significance (p < 0.05) is represented with *. Bars represent mean ± SEM.


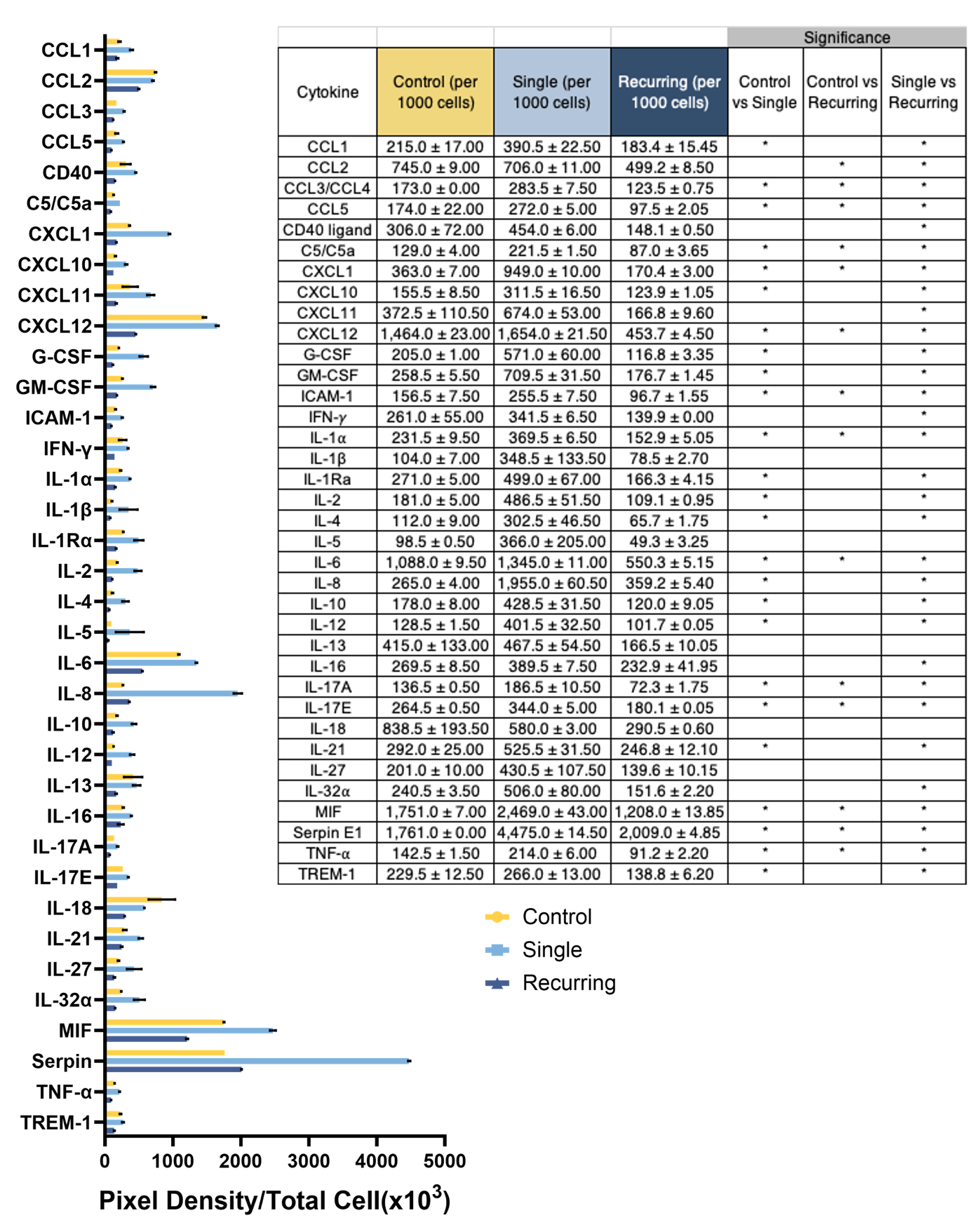


Supplemental Figure 7: Induction method effects senescent secretome. The supernatant from control, single stressed, and recurringly stressed senescent cells was probed for 36 secreted cytokines. Single oxidatively stressed cells secrete a pro-inflammatory secretome compared to the control and recurringly stressed cells.


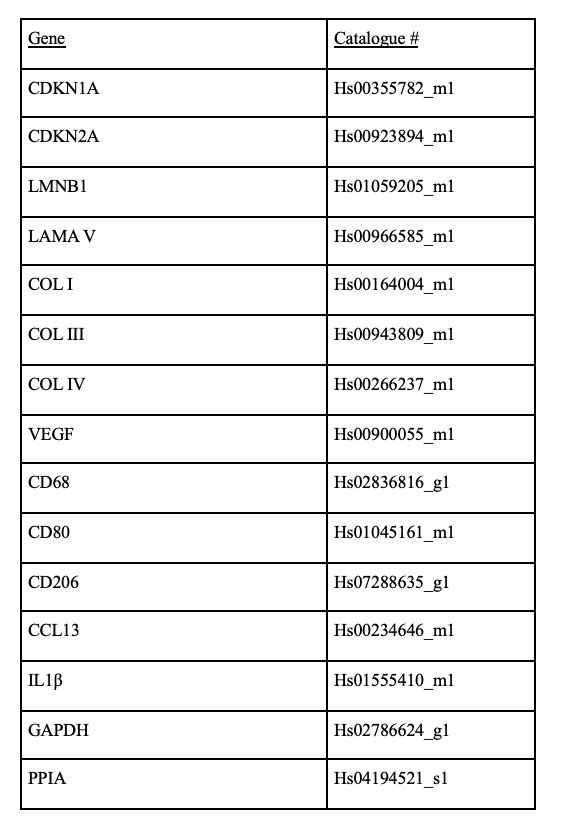


Supplemental Figure 8: All Taqman probes used in this work. All probes were ordered from ThermoFisher.
